# The impact of indole-3-lactic acid on immature intestinal innate immunity and development: a transcriptomic analysis

**DOI:** 10.1038/s41598-021-87353-1

**Published:** 2021-04-13

**Authors:** Wuyang Huang, Ky Young Cho, Di Meng, W. Allan Walker

**Affiliations:** 1 Institute of Agro-Product Processing, Jiangsu Academy of Agricultural Sciences, Nanjing, People’s Republic of China; 2 Department of Pediatrics, Kangnam Sacred Heart Hospital, Hallym University College of Medicine, Seoul, South Korea; 3 Mucosal Immunology and Biology Research Center, Massachusetts General Hospital for Children, Harvard Medical School, 16th Street Building (114-3503), Charlestown, MA 02129 USA

**Keywords:** Cell growth, Cell signalling, Mechanisms of disease, Cell biology, Developmental biology, Gastroenterology, Gastrointestinal diseases, Intestinal diseases, Infant necrotizing enterocolitis

## Abstract

An excessive intestinal inflammatory response may have a role in the pathogenesis of necrotizing enterocolitis (NEC) in very preterm infants. Indole-3-lactic acid (ILA) of breastmilk tryptophan was identified as the anti-inflammatory metabolite involved in probiotic conditioned media from *Bifidobacteria longum* subsp *infantis*. This study aimed to explore the molecular endocytic pathways involved in the protective ILA effect against inflammation. H4 cells, Caco-2 cells, C57BL/6 pup and adult mice were used to compare the anti-inflammatory mechanisms between immature and mature enterocytes in vitro and in vivo. The results show that ILA has pleiotropic protective effects on immature enterocytes including anti-inflammatory, anti-viral, and developmental regulatory potentials in a region-dependent and an age-dependent manner. Quantitative transcriptomic analysis revealed a new mechanistic model in which STAT1 pathways play an important role in IL-1β-induced inflammation and ILA has a regulatory effect on STAT1 pathways. These studies were validated by real-time RT-qPCR and STAT1 inhibitor experiments. Different protective reactions of ILA between immature and mature enterocytes indicated that ILA’s effects are developmentally regulated. These findings may be helpful in preventing NEC for premature infants.

## Introduction

This laboratory has a longstanding interest in examining the effect of colonizing bacteria, particularly probiotics and their metabolites, on the protection of immature intestine from excessive inflammation. The overall goal is to identify nutritional and bacterial factors that prevent necrotizing enterocolitis (NEC). NEC is a devastating intestinal disease of premature infants characterized by excessive inflammation leading to necrosis of the distal ileum and colon^1–3^. The pathogenesis of NEC involves the intestine being invaded by colonizing bacteria which cause intestinal inflammation and ultimately results in intestinal tissue necrosis leading to an overwhelming morbidity and mortality^4,5^. Many studies show that NEC is associated with increased expression of inflammatory cytokines in which IL-1β as a pro-inflammatory stimulus plays an important role in the development of NEC^6–8^.

In this study, quantitative transcriptomic analysis was conducted after an inflammatory stimulus with IL-1β. NEC is believed to be caused by dysbiotic colonization and an aberrant immune response to colonizing bacteria including non-pathologic bacteria^9,10^. Premature infants are at risk for developing this serious gastrointestinal disease due to developmental immaturities of intestinal host defense.

Our previous studies have shown that breastmilk and specific probiotics can produce metabolic products that are anti-inflammatory and help protect the neonate against NEC^11–13^. Administration of probiotic conditioned media (PCM) from *Bifidobacteria longum* subsp *infantis* (*B. infantis*) to several models of NEC confirmed PCM’s protective potential against unrelenting intestinal inflammation^14–16^. The important anti-inflammatory molecule of the secretory fractions of PCM from *B. infantis* is indole-3-lactic acid (ILA), a metabolite of tryptophan in breastmilk after fermentation by *B. infantis*^17^. Sakurai et al. also reported that ILA was the principal tryptophan metabolite produced in *Bifidobacterium* strains, isolated from human infants, which involves immunoregulation and a specific role in host–microbial crosstalk^18^. However, the underlying molecular pathways involved in the protective function against inflammation by ILA in immature enterocytes are as yet unknown. Furthermore, whether ILA has other functions in immature enterocytes remains unclear.

In this study, H4 cells, a well-characterized human fetal primary small intestinal epithelial cell line^19–21^, were used to investigate the molecular mechanisms including the signaling pathway of ILA in protection of immature enterocytes from excessive inflammation. Next generation sequencing (NGS) and quantitative transcriptomic analysis were conducted in H4 cells after an inflammatory stimulus with IL-1β. In addition, the comparison of the anti-inflammatory enterocyte cellular mechanism for ILA’s response between immature and mature enterocytes was conducted in vitro and in vivo. Besides the anti-inflammatory effects of ILA, the molecule has anti-viral potential in response to inflammation and has regulatory effects on immature enterocytes growth. In this study, we also found that the STAT1 cell signaling pathway is involved in IL-1β-induced inflammation in H4 cells and ILA has a regulatory effect on the STAT1 signaling pathway in the prevention of inflammation. Immature and mature enterocytes have been noted to have different reactions upon ILA treatment with or without IL-1β stimulation suggesting that the effects of ILA on enterocytes are developmentally regulated.

## Materials and methods

### Materials and chemicals

Dulbecco’s modified Eagles medium (DMEM), Opti-MEM I medium, MEM nonessential amino acid (NEAA, 100×), glutamine (100×), antibiotic–antimycotic (100×), sodium pyruvate (100 mM), HEPES [4-(2-hydroxyethyl)-1-piperazineethanesulfonic acid] buffer (1 M), Trizol reagent, and the BCA (bicinchoninic acid) Protein Assay Kit were obtained from Thermo Fisher Scientific (Waltham, MA.), Rnase-free Dnase and RNeasy Mini kit were purchased from Qiagen (Frederick, MD.), Fetal bovine serum (FBS) was obtained from Atlanta Biologicals (Flowery, GA) (catalog #: S12450). Humulin R (insulin human recombinant) (100 units/mL) was obtained from Eli Lilly and Company (Indianapolis, IN). Tissue culture plastics were purchased from Fisher Scientific (Pittsburgh, PA). All-in-one cDNA Synthesis SuperMix was purchased from Bimake.com (Houston, TX), and 2× High ROX -Apex qPCR GREEN Master Mix was purchased from Genesee Scientific (EI Cajon, CA). Recombinant human interleukin-1beta (IL-1β), recombined mouse epidermal growth factor (EGF), the enzyme-linked immunosorbent assay (ELISA) kits for human IL-8 and interferon gamma (INFγ) were obtained from R&D Systems (Minneapolis, MN). The LDH (lactate dehydrogenase) Cytotoxicity Detection Kit were bought from Roche Applied Science (Branford, CT). DL-indole-3 lactic acid (ILA), transferrin, sodium selenite, hydrocortisone, and signal transducer and activator of transcription 1 (STAT1) inhibitor fludarabine phosphate (FAP) were purchased from Sigma Aldrich (Natick, MA). All the chemicals and reagents were analytical grade.

### Cell cultures and treatments

H4 cells, a human fetal non-transformed primary intestinal epithelial cell line characterized by our laboratory (IRB 2018-P002987), were used as an in vitro model of the immature intestine^19^. The cells were routinely maintained in DMEM supplemented with 10% FBS (heat-inactivated), 1% NEAA, 2 mM l-glutamine, 1% antibiotic–antimycotic solution, 10 mM HEPES buffer, 1 mM sodium pyruvate and 0.2 units/mL human recombinant insulin. Caco-2 cells were obtained from the American Type Culture Collection (ATCC, Manassas, VA) and cultured in DMEM with 10% FBS, 2 mM l-glutamine, 1% NEAA, 10 mM HEPES buffer, 100 units/mL penicillin and 100 μg/mL streptomycin. Cells were incubated at 37 °C in a 5% carbon dioxide, humidified atmosphere. The cells used for all experiments existed at 80–90% confluence. H4 or Caco-2 cells were seeded in 6-well/12-well plates and incubated with or without 5 μM of ILA for 24 h then stimulated with or without 1 ng/mL of IL-1β for 4 h (for total RNA isolation of the cells) or 24 h (determination of IL8 secretion in the supernatants). Control and IL-1β treated cells were pretreated with dimethyl sulfoxide (DMSO), the solvent used to dissolve ILA.

### Animals and treatments

C57BL/6 mice obtained from Jackson Laboratory were housed in a specific pathogen-free facility. Animals were given water and standard laboratory chow ad libitum. Littermate newborn pups were randomly divided into the control (1× phosphate buffered saline-PBS, pH = 7.4) and ILA (10 μM in 0.1% bovine serum albumin-BSA in 1× PBS) groups. Each pup at P4 (postnatal day 4) received 5 μL of ILA or 0.1% BSA in PBS (control) via gavage feeding once on the first day followed by 7 μL twice per day for 3 days and then 7 μL once on the fifth day. At the end of the experiments the intestinal tissues of ileum and colon were collected and cut into 3-mm pieces for organ culture, meanwhile some intestinal tissues were stored at − 80 °C for total RNA isolation. Organ culture media consisted of Opti-MEM I medium supplemented with 10% FBS, 2 mM l-glutamine, 10 mM HEPES buffer, 0.2 U/mL insulin, and 20 ng/mL recombinant mouse EGF, 5 μg/mL transferrin, 0.06 μM sodium selenite, 200 nM hydrocortisone, and an 1% antibiotic–antimycotic cocktail (100 unit/mL penicillin, 100 μg/mL streptomycin, and 0.25 μg/mL fungizone antimycotic). After 1 h at 37 °C of preincubation, tissues were stimulated with 1 ng/mL of recombinant mouse IL-1β for 2 h. The intestinal tissues were collected and stored at − 80 °C for RNA isolation. The ILA ex vivo experiments were also performed in adult C57BL6 (8–12-week-old male/female, with the weight about 20 g,) mice with the oral feeding of ILA 80–90 μL per mouse per day or the same volume of 0.1% BSA in PBS as the control. The methods of the intestinal tissue culture were the same as described in pup mouse. The weight of C57BL/6J pups and adult mice were shown in Fig. S1.

*Statement* All methods used in animal procedures were carried out in accordance with Massachusetts General Hospital animal use regulations and guidelines with the protocol approved by the Institutional Animal Care and Use Committee of Massachusetts General Hospital (approved protocol number: 2018N000070). The study was carried out in compliance with the Animal Research: Reporting In Vivo Experiments (ARRIVE) guidelines 2.0.

### Total RNA isolation

Trizol/RNeasy hybrid protocol was used for total RNA isolation. H4 or Caco-2 cells were dissolved in 1 mL of Trizol and transferred to a 2 mL Eppendorf tube and incubated at room temperature for 5 min, then 0.2 mL of chloroform was added and the tubes gently shaken for 20 s and then kept at room temperature (RT) for 5 min, followed by a spin at 14,000× rpm at 4 °C for 30 min. The upper layer of the supernatant was transferred to a new tube and the same volume of 70% alcohol was added, followed by the procedure of Qiagen RNeasy Mini Kit for total RNA isolation (with DNase I step to remove remaining DNA) to get total RNA. The total RNA was dissolved in RNase and DNase free water and kept in – 80 °C for later use. Experiments were performed in triplicate. The intestinal tissues (ileum and colon) of pup or adult mice were homogenized in Trizol then the same procedure followed as described above to get the total RNA. The quantity of the extracted RNA was measured by using a Nano Drop ND-1000 spectrophotometer (Thermo Scientific, Wilmington, DE/Rockford, IL, USA). RNA samples with A_260_/A_280_ and A_260_/A_230_ ratios of 1.8–2.0 were used only for further analysis.

### Transcriptomic studies

#### RNA sequencing, identification and functional network analysis of DEGs

The Department of Molecular Biology Bioinformatics Core at Massachusetts General Hospital performed RNA-seq library preparation, sequencing, and read alignment on an Illumina HiSeqTM 2500 (Illumina, San Diego, USA), resulting in approximately 30 million 50 bp reads per sample. After removing low-quality reads with ambiguous nucleotides and sequencing adapters, high-quality reads (clean reads) were mapped in a splice-aware fashion to the human transcriptome reference (hp19/GRCh37.75 assembly) using STAR. Differentially expressed genes (DEG) between the groups were identified with edgeR method, after normalization with log2 counts per million (log CPM). False discovery rate (FDR) < 0.05 and |log2-Fold Change (FC)| ≥ 0.4 were considered as a cutoff criterion for DEGs mRNA selection. Hierarchical clustering analysis was conducted from triplet experiment samples with IL-1β and ILA under the IL-1β stimulation before down-stream analysis. A Venn diagram was constructed to show the overlapping expressed genes in the control and groups pretreated with ILA (5 μM) for 24 h in the absence or presence of IL-1β stimulation (1 ng/mL for 4 h).

A multi-expression viewer (MeV) online tool was used to perform the functional enrichment analyses for Kyoto Encyclopedia of Genes and Genomes (KEGG) pathway, and Gene ontology (GO) terms for biological processes (BP), cellular components, and molecular function (MF) on the DEGs at a threshold of an FDR *P* value less than 0.05.

#### Construction of protein–protein interaction (PPI) network and functional enrichment analysis map

A PPI network, corresponding to the upregulated DEGs associated with ILA, was constructed by Cytoscape software with the reference of the Search Tool for the Retrieval of Interacting Genes (STRING) database. The top 10 upregulated genes were identified based on the topological parameter of the PPI network with a threshold of degree 6, using a Cytoscape NetworkAnalyzer plug-in. Functional enrichment analyses for the top 10 upregulated genes were visualized with Cytoscape ClueGo plug-in with Bonferroni corrected *P*-value significance < 0.05. The top 10 upregulated genes associated with ILA were validated for their coexpression with other interacting genes using a FpClass web-based tool which gave the gene co-expression score and network topology score of each gene.

#### Real-time quantitative reverse transcription PCR (qRT-PCR) analysis

RNA was reverse transcribed with an All-in-one cDNA Synthesis SuperMix kit. The cDNA was amplified using 2× High ROX Apex qPCR GREEN Master Mix. The genes, determined by quantitative RT-PCR in this study, were shown in Table S1 in which the primer sequencing of these genes are also included. qRT-PCR experiment was carried out in a 96-well plate with an Applied Biosystems StepOnePlus Real-Time PCR system (Thermo Fisher Scientific Inc., San Diego, CA). GAPDH primers were amplified in all samples to represent the housekeeper gene. Triplicate cDNA samples were amplified with the primers, mean threshold cycle (Ct) values of each transcript were normalized by subtracting the mean Ct value for the GAPDH transcript of that sample. Relative gene expression data was analyzed using the 2^−ΔΔCt^ method. All data were expressed as fold increase over the corresponding control.

### LDH cytotoxicity assay

A lactate dehydrogenase (LDH) cytotoxic assay was performed to determine the enterocytes integrity and viability during the course of the experiments. This assay is a colorimetric assay based on the measurement of LDH activity released from the cytosol of damaged cells into the supernatant. An LDH Assay Kit was used following the manufacturer’s instructions. The optical density (OD) of LDH was read by a spectrophotometer at 490 nm. The cell culture medium was used as a blank control while the supernatant from the cells treated with 2% of the triton x-100 was used as a positive control. The results are expressed as a percentage of the maximum LDH release. Cytotoxin (%) = (OD_sample_ − OD_blank_)/(OD_positive control_ − OD_blank_) (%).

### ELISA analysis

IL-8 or INFγ secretion into the cell culture supernatants was determined by ELISA kits. The total cell protein in each well was detected by using BCA Protein Assay Kit. The assay procedure was employed according to the manufacturer’s protocols. Absorbance at 450 nm was measured for Elisa and at 562 nm was measured for protein by using a Multiskan Go microplate spectrophotometer (Thermo Fisher Scientific Inc., San Diego, CA).

### H4 cells subjected to a STAT1 inhibitor

To investigate the function of STAT1 in ILA prevention of IL-1β-induced inflammation in immature enterocytes, H4 cells were pretreated with STAT1 inhibitor FAP^22^ at the dosage of 0.01, 0.1, and 1 μg/mL (dissolved in DMSO) for 30 min then treated with ILA for 24 h, following by exposure to 1 ng/mL of recombinant human IL-1β for 4 h (the cells were collected for RNA isolation) or 24 h (the supernatant was collected for an IL-8 Elisa assay and an LDH assay).

### Statistical analysis

All data are presented as the mean ± standard error of the mean (SEM). The sequencing data were processed with RStudio, Version 4.0.2 (RStudio team (2020). RStudio: Integrated Development for R. RStudio, PBC, Boston, MA URL http://www.rstudio.com/). MeV web-based tool (http://mev.tm4.org), STRING (version 11.0), FpClass web based tool (http://dcv.uhnres.utoronto.ca/FPCLASS) and Cytoscape (version 3.8.0). Statistical significance was declared at a *P* value < 0.05 and resulting *P* values were adjusted for multiple testing with an FDR method. Figures were obtained by using GraphPad Prism Version 7 (GraphPad Software, Inc., CA, USA). One-way or two-way analysis of variance (ANOVA) was used to compare the mean of multiple groups. Repeated test was used wherever applicable. *p* < 0.05 were considered significant differences (*p < 0.05, **p < 0.01, ***p < 0.001).

## Results

### Quantitative transcriptomic analysis of the effects of ILA on immature human enterocytes (H4 cells)

#### Identification and functional enrichment analysis of DEGs

**Figure 1:**
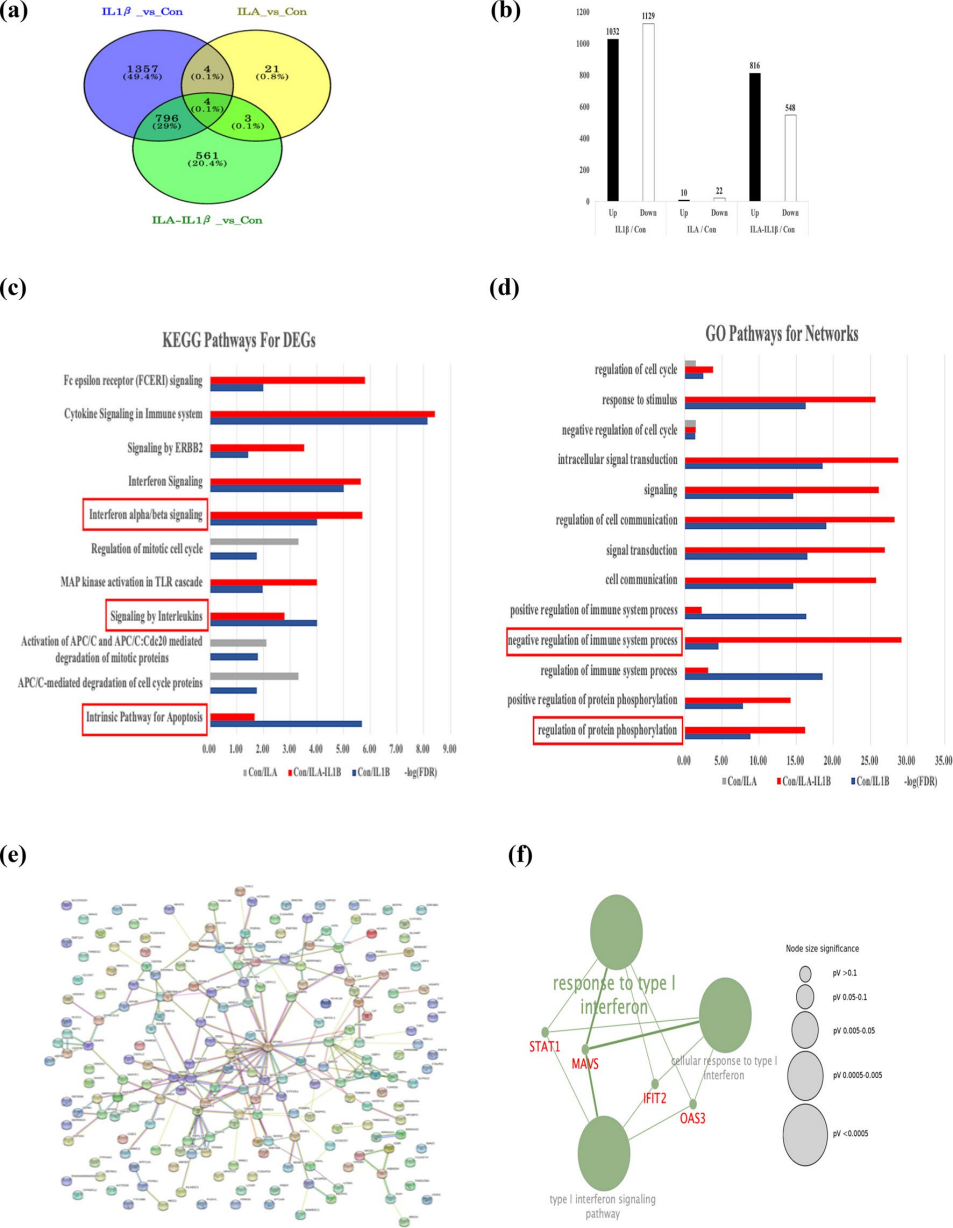
The characteristics of the differentially expressed genes (DEGs), major enriched KEGG pathways involved in immune response for DEGs, and Protein–protein interaction network. DEGs between the groups were identified with edgeR method, using RStudio, Version 4.0.2 (RStudio team (2020). RStudio: Integrated Development for R. RStudio, PBC, Boston, MA URL http://www.rstudio.com/). DEGs in H4 cells that were defined as those whose expression values with a set of |log2-Fold Change| ≥ 0.4 and FDR < 0.05. (**a**) A Venn diagram showing the overlapping DEGs in H4 cells of the control and groups pretreated with ILA in the absence or presence of IL-1β stimulation. (**b**) Histogram showing the total number of up and downregulated DEGs in the H4 cells of the control and groups pretreated with ILA in the absence or presence of IL-1β stimulation. The major enriched KEGG pathways involved in immune response for DEGs (**c**) and major enriched GO terms involved in the immune response for protein–protein networks (**d**) in the upregulated genes associated by IL-1β, ILA-IL-1β, and ILA compared with the control, using the STRING tool. Protein–protein interaction network plot for upregulated genes induced by ILA between the IL-1β and ILA-IL-1β group, using the STRING online tool (**e**). GO enrichment analysis map for the top 10 upregulated genes induced by ILA, using Cytoscape ClueGo plug-in (**f**). DEGs, differentially expressed genes; FDR, false discovery rate; KEGG, Kyoto Encyclopedia of Genes and Genomes; STRING, Search Tool for the Retrieval of Interacting Genes; GO, gene ontology.

H4 cells were pretreated with or without ILA before IL-1β stimulation to explore if ILA could protect immature enterocytes from inflammation. In order to find differentially expressed genes (DEG) between the groups, quantitative transcriptomic analysis was conducted. A Venn diagram (Fig. 1a) showing the number of DEG genes identified specifically in the IL-1β, ILA, and ILA-IL-1β groups by comparison with the control were 1357, 21, and 561, respectively. The number of common DEG genes in all groups by comparison with the control was 4 (0.1%) (Fig. 1a) suggesting different treatments can induce specific gene changes, in common with each other and in common with all gene changes. The results of hierarchical clustering analysis showed that IL-1β-sample 2 and ILA-IL-1β-sample 3 displayed a deviation and thus were removed (Fig. S2). Figure 1b showed a total number of 1032, 10, and 816 DEGs were upregulated, while 1129, 22, and 548 DEGs were downregulated in association with IL-1β, ILA, and ILA-IL-1β groups in comparison with the control, respectively.

The functional enrichment analysis of the MeV web-based tool identified 55 overlapping KEGG pathways for DEGs in the IL-1β, ILA, and ILA-IL-1β groups compared with the control (Table S2). The major KEGG pathways involved in the immune response for those pathways were shown in Fig. 1c. Among them, the FDR significance of “interferon alpha/beta signaling” in the ILA-IL-1β treated group was higher than that in the IL-1β treated group compared with the control, and the FDR significance of “signaling by interleukins” and “intrinsic pathway for apoptosis” in the IL-1β treated group was higher than that in the ILA-IL-1β treated group compared with the control (Fig. 1c).

#### Protein–protein interaction (PPI) network construction and the identification of the top 10 upregulated genes induced by ILA

PPI networks significantly enrich pathways associated with the common functions. STRING tool was used to construct the PPI network for the upregulated genes associated with IL-1β, ILA, and ILA-IL-1β groups compared with the control. The overlapping GO terms for the PPI networks in the IL-1β, ILA, and ILA-IL-1β groups compared with the control were 745 terms (unshown data). Major enriched gene ontology (GO) terms associated with the immune response for those networks were shown in Fig. 1d. Among them, the FDR significances of “negative regulation of immune system process” and “regulation of protein phosphorylation” were higher in the ILA-IL-1β treated group than in the IL-1β only group compared with the control (Fig. 1d). The PPI network for upregulated genes induced by ILA between the IL-1β and ILA-IL-1β comparison groups were built by the STRING tool (Fig. 1e) and subsequently the topological parameters for the network were analyzed by the Cytoscape NetworkAnalyzer plug-in. The top 10 upregulated genes induced by ILA were identified based on the topological parameters of the PPI network at a threshold of degree 6 (Table 1).

**Table 1:** The top 10 upregulated genes in the IL-1β/ILA-IL-1β group identified based on the topological parameters at a threshold of degree 6 in the protein–protein interaction network, using the Cytoscape NetworkAnalyzer plug-in.

Gene name	Degree	Betweenness centrality	Closeness centrality
EP300	23	0.575512787	0.408496732
STAT1	12	0.167226467	0.327225131
DICER1	10	0.171482121	0.332446809
HUWE1	9	0.179622245	0.337837838
DDX6	9	0.080730149	0.296208531
PIK3R1	7	0.158115054	0.329815303
MED13L	7	0.09930223	0.322164948
MAVS	7	0.05476965	0.277777778
OAS3	7	0.018437946	0.262054507
IFIT2	7	0.002846548	0.262054507

#### The functional enrichment analysis map and the identification of key genes

Discovering functional modules in a PPI network is very important for understanding the organization and function of the related biological system. The functional enrichment analysis map was conducted on the top 10 upregulated genes induced by ILA, using the Cytoscape ClueGo plug in (Fig. 1f). Nodes with Bonferroni corrected p-value significance < 0.05 are expressed, colored with group and those size are of correlated significance (Fig. 1f). The kappa coefficient score, defining term-term interrelations and functional groups based on shared genes between terms, is used for edges (Fig. 1f). The edges are expressed for a kappa score greater than 0.4 and those thickness are correlated with the score (Fig. 1f). We identified each group with 3 GO terms for the biological process (BP); response to type I interferon, cellular response to type I interferon, and type I interferon signaling (Fig. 1f). These GO terms included 4 common genes (STAT1, MAVS, IFIT2, OAS3) which could be the key genes induced by ILA in comparison with IL-1β and ILA-IL-1β groups (Fig. 1f).

FpClass tool was used to validate the top 10 upregulated genes for their co-expression with other interacting genes. The partner scores of those genes and their partner genes which have the highest total score and gene co-expression scores in the FpClass analysis are presented in Table 2. Among these genes, STAT1 was predicted to have the highest total score of 0.9429, giving evidence of the strong interaction with the partner gene CXCL10 (Table 2).

**Table 2:** Partner scores of the top 10 upregulated genes associated with ILA between the IL-1β and ILA-IL-1β groups analyzed by the FpClass tool.

Genes	Predicted partner gene	Total score	Gene co-expression score	Network topology score
STAT1	CXCL10	0.9429	0.588	0.1404
EP300	SMAD4	0.9429	0.0285	0.2896
DICER1	EIF2C3	0.8826	0.0954	0
HUWE1	TP53	0.8826	0.026	0.6567
DDX6	EIF2C2	0.8826	0.0196	0.2943
PIK3R1	PIK3CA	0.9429	0.0291	0.2051
MED13L	MED13	0.8826	0.1926	0.4343
MAVS	IRF3	0.8826	0.0881	0.612
OAS3	MX1	0.8826	0.8779	0
IFIT2	OASL	0.8826	0.8799	0

### Anti-inflammatory potential of ILA

#### A different anti-inflammatory potential of ILA in immature and mature human enterocytes

**Figure 2:**
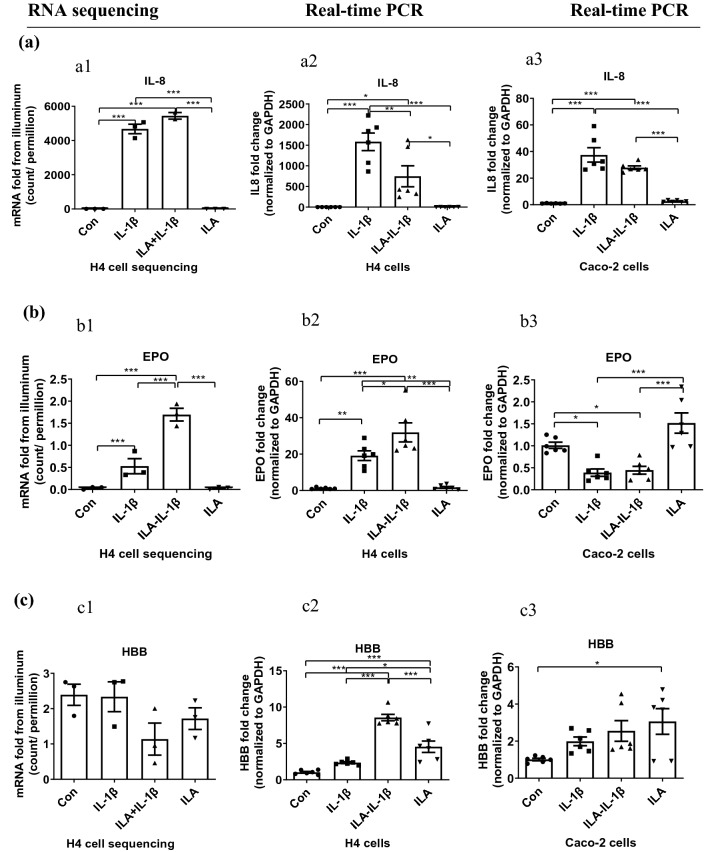
The anti-inflammatory potential of ILA is different in immature and mature enterocytes. (**a**) ILA inhibited IL-1β-induced IL-8 mRNA expression in both H4 (a2) and Caco2 (a3) cells by real time PCR, but not by the Illumina method (a1). (**b**) The effects of ILA on anti-NEC inflammatory factor EPO mRNA expression in H4 cells (b1 and b2) and Caco2 cells (b3). (**c**) The impact of ILA on anti-inflammatory factor HBB mRNA expression in response to IL-1β in H4 (c1, c2) and Caco2 (c3) cells. RNA sequencing analysis in H4 cells by using Illumina assay (a1, b1, and c1), the validation of RNA sequencing was conducted by qRT- PCR in H4 cells (a2, b2, and c2) and compared with Caco-2 cells (qRT-PCR)(a3, b3, and c3). H4 or Caco-2 cells were pretreated with ILA before IL-1β stimulation the mRNA levels were detected by RNA sequencing or real time PCR. Data are represented as the mean ± SEM (n = 3 for RNA sequencing, n = 6 for q-RT-PCR). Real time PCR represented one of two independent experiments. One-way ANOVA and Tukey post hoc tests were used for statistical analysis. Differences were considered significant at *p < 0.05, **p < 0.01, *** *p* < 0.001. IL-8, interleukin 8; EPO, erythropoietin; HBB, hemoglobin subunit beta.

The validation of RNA sequencing was conducted by real-time quantitative reverse transcription PCR (qRT-PCR) in H4 cells and most of the detected gene expressions were consistent between real-time qRT-PCR and RNA-sequencing Illumina analysis (Fig. S3). IL-1β is a proinflammatory cytokine playing a role in multiple inflammatory-associated disorders, which can induce excessive inflammatory chemokine IL-8. In this study, IL-8 mRNA expression in both H4 cells and Caco-2 cells was greatly increased after 1 ng/mL of IL-1β stimulation for 4 h (*p* < 0.001, Fig. 2a2,a3). Real-time qRT-PCR results confirmed the difference in anti-inflammatory potential of ILA in human immature enterocytes (H4 cells) and mature enterocytes (Caco-2 cells). Pretreatment with 5 μM of ILA for 24 h significantly inhibited IL-1β-induced IL-8 mRNA expression in H4 cells (*p* < 0.01, Fig. 2a2), while the decrease of IL-1β-induced IL-8 mRNA expression produced by ILA in Caco-2 cells was not significant (*p* > 0.05, Fig. 2a3). It was strange that there was no difference of IL-8 mRNA expression between IL-1β and ILA-IL-1β groups from RNA sequencing by Illumina assay (Fig. 2a1). Illumina assay results are representative as the number of reads per million count and qRT-PCR results are representative as cycle threshold (CT) normalized to glyceraldehyde 3-phosphate dehydrogenase (GAPDH) suggesting that the two methods may not get the consistent results in 100 percent of circumstances, but results from the two methods are mostly consistent (Fig. S3). The above results indicated that ILA exhibited an anti-inflammatory response to IL-1β in human immature enterocytes. In H4 cells, ILA pretreatment also reduced the IL-1β-induced proinflammatory mediator plate-activating factor-1 (PAF1) and its receptor (PAFR) mRNA expression (Fig. S7a,b), reduced the IL-1β-induced TNF receptor associated factor2 (TRAF2) mRNA expression (Fig. S7d) and increased IL6 mRNA expression (Fig. S7c), but had no effect on IL10 mRNA expression (Fig. S7e).

Erythropoietin (EPO), the human milk factor, has been shown to protect endothelial cell–cell and blood–brain barriers and can specifically preserve intestinal barrier function under conditions of inflammatory stress, and is therefore a novel target gene associated with the immune response^23^. Here EPO mRNA expression was increased in H4 cells, while decreased in Caco-2 cells after 1 ng/mL of IL-1β stimulation (*p* < 0.05, Fig. 2b). Pretreatment with 5 μM of ILA for 24 h could further increase the EPO mRNA expression in H4 cells stimulated by IL-1β (*p* < 0.001, Fig. 2b1 and *p* < 0.05, Fig. 2b2). However, no difference existed between the control and ILA group. In Caco-2 cells, although pretreatment with ILA did not change EPO mRNA expression under inflammatory stress by IL-1β, ILA group’s EPO mRNA expression was significantly higher than IL-1β and ILA-IL-1β groups (*p* < 0.001, Fig. 2b3).

The hemoglobin subunit beta (HBB), a subunit that is involved in oxygen transport from the lung to various peripheral tissues, can be upregulated by EPO^24^ whose C-terminal region (112–147) is a broadly active antimicrobial innate immune defense peptide^25^. IL-1β stimulation didn’t significantly change HBB mRNA expression in both H4 cells and Caco-2 cells. (*p* > 0.05, Fig. 2c). Pretreatment with 5 μM of ILA for 24 h did pronouncedly increase the HBB mRNA expression in H4 cells with or without IL-1β stimulation (*p* < 0.001, Fig. 2c2). However, no difference existed among all the groups according to RNA sequencing Illumina analysis (*p* > 0.05, Fig. 2c1). There was a significant difference between qRT-PCR and RNA-sequencing Illumina assay when comparing the HBB gene expression changed ratio (Fig. S3). This might be because HBB is common in adult humans, but its expression is too low to be stable in immature enterocytes. In Caco-2 cells, ILA could greatly increase HBB mRNA expression without inflammatory stress by IL-1β (*p* < 0.05, Fig. 2c3).

#### A different anti-inflammatory potential of ILA in immature versus mature mouse intestine

**Figure 3:**
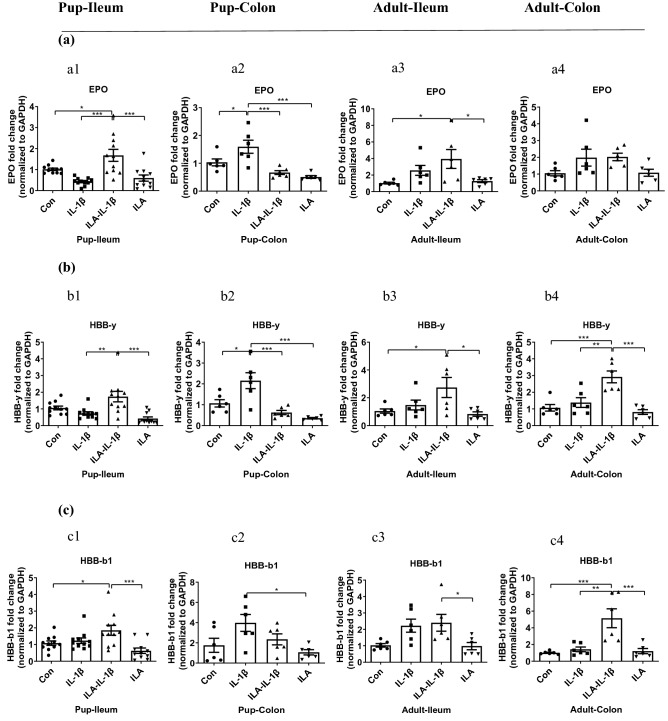
ILA affects potential anti-inflammatory gene expression in a range different and age different manners in mice intestine. (**a**) EPO, (**b**) HBB-y (beta-like embryonic chain), and (**c**) HBB-b1(adult major chain) mRNA gene expression level of qRT PCR in ileum (a1, b1, and c1) and colon (a2, b2, and c2) of pups; and ileum (a3, b3, and c3) and colon (a4, b4, and c4) of adults. Pup or adult mice were fed with or without ILA, whose intestinal tissues were incubated with or without IL-1β stimulation. Data are represented as the mean ± SEM (n = 6). One-way ANOVA and Tukey post hoc tests were used for statistical analysis. Differences were considered significant at *p < 0.05, **p < 0.01, *** *p* < 0.001. EPO, erythropoietin; HBB-y, hemoglobin Y, beta-like embryonic chain; HBB-b1, hemoglobin, beta adult major chain).

To further investigate if ILA has similar anti-inflammatory effects in vivo, C57BL/6 littermate newborn pups and more than 8-weeks old adult mice were orally administrated with ILA followed by intestinal tissue exposure to IL-1β stimulation in vitro. Potential anti-inflammatory genes EPO, HBB-y (beta-like embryonic chain), and HBB-b1(adult major chain) mRNA fold change was determined by real-time qRT-PCR in ileum and colon tissues. The results show ILA affected the gene expression by a region-dependent and an age-dependent manner in mouse intestine. Pretreatment with ILA significantly increased the EPO mRNA expression in pup-ileum stimulated by IL-1β (*p* < 0.001, Fig. 3a1), but decreased the EPO mRNA expression in pup-colon stimulated by IL-1β (*p* < 0.001, Fig. 3a2). However, ILA did not change EPO mRNA expression in adult-ileum/colon with or without IL-1β stimulation (Fig. 3a3,a4).

With regard to hemoglobin beta-like embryonic chain, the change of HBB-y mRNA expression in the ileum and colon of pups was similar to that of EPO (Fig. 3b1,b2). However, there was no significant change in the adult major chain HBB-b1 in pups (Fig. 3c1,c2). Pretreatment with ILA could significantly increase both HBB-y and HBB-b1 mRNA expression in the colon of pups and adults with inflammatory stress by IL-1β (*p* < 0.01, Fig. 3b4,c4), but not in the ileum (Fig. 3b3,c3).

### Anti-viral potential of ILA

#### A different anti-viral potential of ILA in immature versus mature human enterocytes

**Figure 4:**
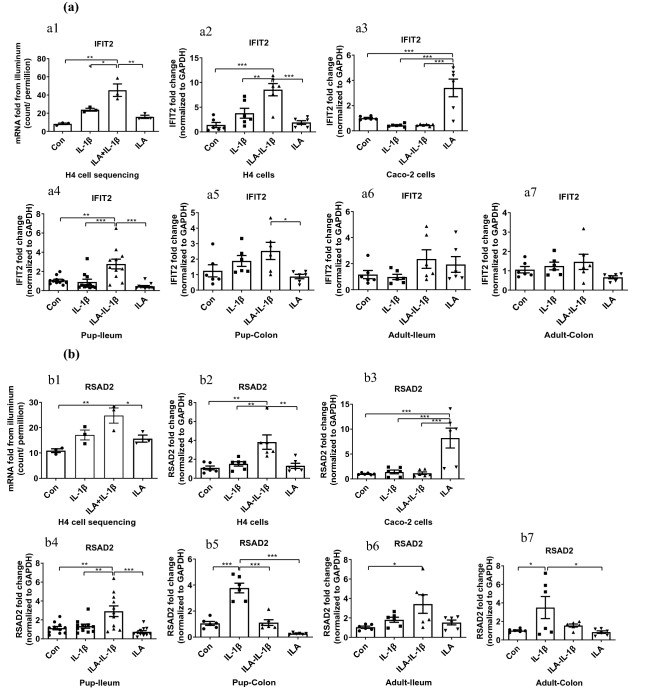
ILA increased anti-viral/related gene expression in immature human enterocytes and fetal mouse ileum in response to IL-1β. RNA sequencing and qRT-PCR were used. (**a**) IFIT2 and (**b**) RSAD2 mRNA sequencing analysis using Illumina (a1 and b1) and gene expression level of qRT PCR in H4 cells (a2 and b2) and Caco-2 cells (a3 and b3); in ileum (a4 and b4) and colon (a5 and b5) of pups; and ileum (a6 and b6) and colon (a7 and b7) of adults. H4 or Caco-2 cells were pretreated with ILA before IL-1β stimulation. Pup or adult mice were fed with or without ILA, whose intestinal tissues were incubated with or without IL-1β stimulation. Data are represented as the mean ± SEM (n = 3–6 for cells in repeated experiment, n = 6 for mouse). One-way ANOVA and Tukey post hoc tests were used for statistical analysis. Differences were considered significant at *p < 0.05, **p < 0.01, ***p < 0.001. IFIT2, interferon-induced protein with tetratricopeptide repeats 2; RSAD2, radical S-adenosyl methionine domain containing 2.

The PPI network showed ILA was not only involved in the “negative regulation of immune system process” but was also involved in “interferon alpha/beta signaling” which indicated ILA had anti-viral potential. Interferon-induced protein with tetratricopeptide repeats 2 (IFIT2), one of the top 10 upregulated genes induced by ILA, is an anti-viral protein preventing tumor progression and regulating viral replication^26^. Radical S-adenosyl methionine domain containing 2 (RSAD2), also an interferon-inducible virus inhibitory protein, modulates cellular metabolic pathways essential for viral replication and/or cell proliferation and survival^27^. So IFIT2 and RSAD2 gene changes by ILA were investigated. IFIT2 and RSAD2 mRNA sequencing analysis using Illumina (Fig. 4a1,b1) and gene expression level of qRT PCR in H4 cells (Fig. 4a2,b2) was almost identical. ILA pretreatment enhanced the IFIT2 and RSAD2 mRNA expression in H4 cells stimulated by IL-1β (*p* < 0.01, Fig. 4a2,b2), suggesting its anti-viral potential in the immature human enterocyte during inflammatory stress. In Caco-2 cells, IFIT2 and RSAD2 mRNA expression in the ILA group was considerably higher than those of the other three groups (*p* < 0.001, Fig. 4a3,b3), while their values of both IL-1β and ILA-IL-1β groups were very low suggesting ILA’s anti-viral potential in mature human enterocytes without inflammatory stress which was altered by IL-1β.

#### A different anti-viral potential of ILA in immature and mature mouse intestine

In a manner similar to the anti-inflammatory effects, ILA also affected anti-viral genes IFIT2 and RSAD2 mRNA expression in a region-dependent and an age-dependent manner in mouse intestine. ILA pretreatment significantly increased the IFIT2 and RSAD2 mRNA expression in pup-ileum stimulated by IL-1β (*p* < 0.001, Fig. 4a4 and *p* < 0.001, Fig. 4b4), but decreased the RSAD2 mRNA expression in pup-colon stimulated by IL-1β (*p* < 0.001, Fig. 4b5). ILA pretreatment did not significantly change IFIT2 mRNA expression in IL-1β stimulated pup-colon. However, the ILA-IL-1β group expressed more IFIT2 mRNA than the ILA group (*p* < 0.05, Fig. 4a5). For mature mouse intestine, ILA had no anti-viral effect. There was no significant difference in IFIT2 mRNA expression in the ileum and colon of adult mice among the four groups (*p* > 0.05, Fig. 4a6,a7). The RSAD2 mRNA expression of the ILA-IL-1β group in adult-ileum was the highest while the IL-1β group’s in adult-colon was the highest among the four treatments (*p* < 0.05 *vs* Control, Fig. 4b6,b7).

### Cell development potential with ILA

#### A different cell development potential with ILA in immature versus mature human enterocytes

**Figure 5:**
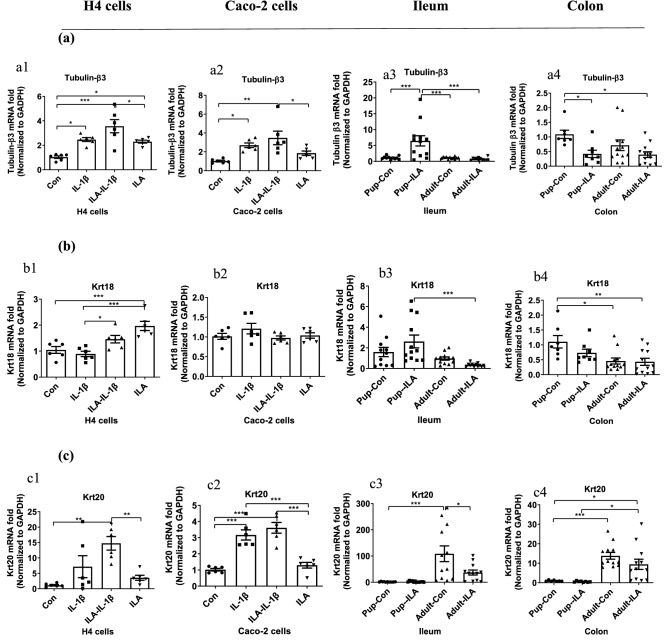
ILA regulated cell development/related gene expression in vitro and in vivo (**a**) Tubulin-β3 and (**b**) Krt18 (embryonic keratin), and (**c**) Krt20 (adult keratin) mRNA gene levels were determined by qRT PCR in H4 cells (a1, b1, and c1), Caco-2 cells (a2, b2, and c2), and ileum (a3, b3, and c3) and colon (a4, b4, and c4) of pups and adults. H4 or Caco-2 cells were pretreated with ILA before IL-1β stimulation. Pup or adult mice were fed with or without ILA. Data are represented as the mean ± SEM (n = 6 for cells and adult mice in repeated experiment, n = 11–12 for pup mice). One-way ANOVA and Tukey post hoc tests were used for statistical analysis. Differences were considered significant at *p < 0.05, **p < 0.01, *** *p* < 0.001.

To investigate the effects of ILA on cell growth in vitro and in vivo, some cell shape and development-associated genes were also determined by real-time qRT-PCR (Figs. 5, S4), including cytoskeleton protein Tubulin-β3, embryonic keratin Krt18, adult keratin Krt20, protein-coding gene cytidine deaminase CDA, and cell proliferation related protein Ki67. ILA upregulated cell development related gene expression in the human immature enterocytes. Pretreatment with ILA directly increased Tubulin-β3 mRNA expression in H4 cells (*p* < 0.05 *vs* Control, Fig. 5a1), but not in Caco-2 cells (Fig. 5a2). IL-1β stimulation also increased Tubulin-β3 mRNA expression but had no significant change with ILA pretreatment in both H4 cells and Caco-2 cells.

In addition, ILA could increase embryonic keratin Krt18 mRNA expression levels in H4 cells with or without IL-1β stimulation (*p* < 0.05, Fig. 5b1). However, Krt18 levels in Caco-2 cells were low and had no change within the four groups (Fig. 5b2). In contrast, adult keratin Krt20 mRNA expression levels in Caco-2 cells were high, which were upregulated by IL-1β stimulation (*p* < 0.001, Fig. 5c2). ILA could not regulate adult Krt20 mRNA expression both in H4 cells or Caco-2 cells (Fig. 5c1,c2). In addition, ILA had no significant effects on the CDA gene (Fig. S4a), which encodes the enzyme, cytidine deaminase, responsible for maintaining the cellular pyrimidine pool.

#### A different cell development potential with ILA in immature versus mature mouse intestine

ILA also affected the cell shape and development-associated genes in a region-dependent and an age-dependent manner in mouse intestine. ILA increased Tubulin-β3 mRNA expression in pup-ileum (*p* < 0.001, Fig. 5a3), but decreased it in pup-colon (*p* < 0.05, Fig. 5a4). In both ileum and colon of adult mice, the decrease of Tubulin-β3 mRNA expression induced by ILA was also significant when compared to its control by *t*-test statistical analysis (Fig. S4b).

Embryonic Krt18 mRNA expression levels in the ileum and colon of pups were higher than those in adults, while adult Krt20 mRNA expression levels in adult-ileum and adult-colon were much higher than those in pups (Fig. 5b3,b4,c3,c4). In organ culture experiments, after intestinal tissues were incubated with or without IL-1β for 2 h, Krt18 mRNA expression in the colon was increased by ILA treatment for pups (*p* < 0.05, Fig. S4c1) but decreased for adults (*p* < 0.001, Fig. S4c2). IL-1β-induced Krt20 mRNA expression in adult-ileum was downregulated by ILA treatment (*p* < 0.01, Fig. S4c4) which was not significant in pup-ileum (Fig. S4c3). The effects of ILA on the embryonic and adult keratin gene were different among pups versus adults which was consistent with the results of hemoglobin genes HBB-y and HBB-b. Moreover, ILA affected the Ki67 (a nuclear protein associated with cellular proliferation) gene expression in immature and mature mouse intestine differently (increase in pup-ileum, but decrease in adult-ileum, Fig. S4d).

### Anti-inflammatory effects of ILA by the STAT1 signaling pathway

#### ILA inhibited the IL-1β-induced inflammatory response by regulating the STAT1 signaling pathway in immature human enterocytes

**Figure 6:**
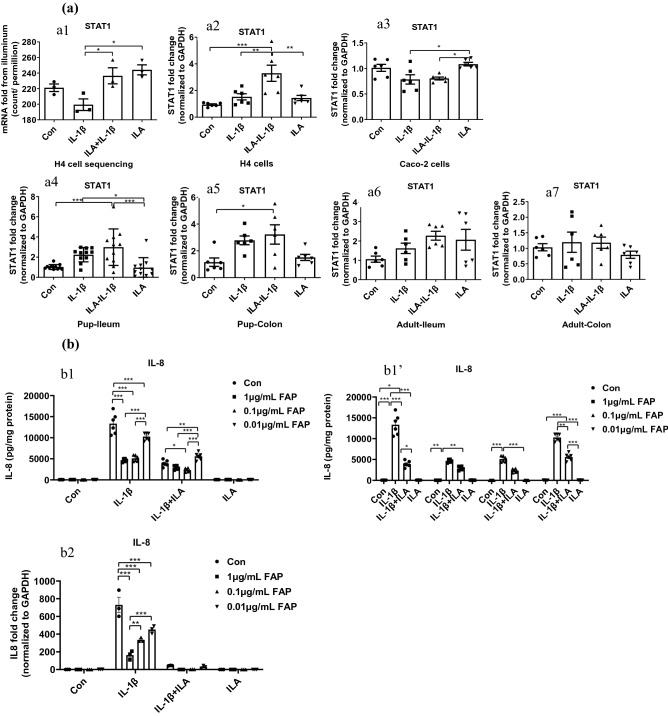
ILA inhibited the IL-1β-induced inflammatory response by regulating STAT1 signaling pathway. (**a**) STAT1 mRNA level analyzed by RNA sequencing using Illumina (a1) and validated by qRT PCR in H4 cells (a2). STAT1 mRNA expression in Caco-2 cells (a3), pup-ileum (a4) and colon (a5), adult ileum (a6) and colon (a7) were determined by qRT-PCR; (**b**) The different concentration of STAT1 inhibitor FAP decreased IL-1β-induced IL-8 secretion (b1, b1′) and mRNA expression (b2) in H4 cells. H4 or Caco-2 cells were pretreated with ILA before IL-1β stimulation. Pup or adult mice were fed with or without ILA for 5 days, intestinal tissues (organ culture) were incubated with or without IL-1β. Different doses of STAT1 inhibitor FAP were pretreated 30 min before ILA treatment for 24 h then exposed to IL-1β. The secretion of IL-8 into the cell culture supernatant was determined by ELISA. Data are represented as the mean ± SEM (n = 3–6 for cells in repeated experiment, n = 6 for mouse). One-way and Two-way ANOVA and Tukey post hoc tests were used for statistical analysis. Differences were considered significant at *p < 0.05, **p < 0.01, *** *p* < 0.001. STAT1, signal transducer and activator of transcription 1; FAP, fludarabin phosphate, STAT1 inhibitor.

Signal transducer and activator of transcription 1 (STAT1), one of the top 10 upregulated genes induced by ILA with the highest total score (Table 2) and one of four key genes according to GO enrichment analysis map (Fig. 1e,f), had a key role in many gene expressions that cause survival of the cell, viability or pathogen response^28^. Therefore, we speculated that ILA inhibited the IL-1β-induced inflammatory response by regulating the STAT1 signaling pathway. STAT1 mRNA levels were analyzed by RNA sequencing using Illumina and validated by qRT PCR in H4 cells. ILA pretreatment enhanced the STAT1 mRNA expression in H4 cells stimulated by IL-1β (*p* < 0.05 (Fig. 6a1,a2). The change trend of STAT1 mRNA expression in the ileum and colon of pups was similar to that in H4 cells with the highest levels in ILA-IL-1β group, but the change by ILA to IL-1β was not significant (Fig. 6a4,a5). In Caco-2 cells, STAT1 mRNA expression of the ILA group was pronouncedly higher than those of the IL-1β-stimulated two groups (*p* < 0.05, Fig. 6a3). However, no significant change was found in the ileum and colon of adult mice.

#### The STAT1 inhibitor FAP enhanced the effects of ILA anti-inflammation

To prove the hypothesis that the ILA-inhibiting inflammatory response was affected by the STAT1 signaling pathway, H4 cells were pretreated with different doses of STAT1 inhibitor FAP (1, 0.1, and 0.01 μg/mL) for 30 min before ILA treatment for 24 h and then exposed to IL-1β. The LDH assay confirmed that various doses of FAP were not cytotoxic (Fig. S5a). The results showed that STAT1 inhibitor FAP greatly decreased IL-1β-induced IL-8 secretion in H4 supernatant (*p* < 0.001, Fig. 6b1) and IL-8 mRNA expression in H4 cells (*p* < 0.001, Fig. 6b2) in a dose-dependent manner. High doses of FAP further decreased the IL-1β-induced IL-8 secretion and enhanced the effects of ILA anti-inflammation suggesting that the STAT1 signaling pathway was protecting H4 cells from IL-1β-induced inflammation but also inhibited the anti-inflammatory effect of ILA since the difference between IL-1β and ILA-IL-1β groups was lost (Fig. 6b1ʹ). However, no change was found on the INFγ secretion in H4 supernatant (Fig. S5b), and FAP decreased INFγ and CYP1A1 mRNA expression in H4 cells (Fig. S5c,d). In addition, EPO, Tubulin-β3, Krt20, RSAD2 mRNA expression levels at the dose of 0.1 μg/mL of FAP pretreatment was consistent with the function of STAT1 (Fig. S6). STAT1 inhibitor FAP confirmed the inhibition of ILA to IL-1β (no significant difference between IL-1β and ILA-IL-1β groups), but directly enhanced the ILA anti-inflammatory potential (the highest level in ILA group). These results further confirmed the key role of the STAT1 gene in regulating anti-inflammatory response.

## Discussion

Necrotizing enterocolitis (NEC) is an inflammatory bowel necrosis that primarily afflicts premature infants after the initiation of enteral feeds. It is thought that NEC is associated with dysbiosis of the gut microbiome^29^. Emerging evidence suggests that small-molecule metabolites derived from bacterial breakdown of a variety of dietary nutrients confer a wide array of host benefits, including inhibition of inflammation^30^. Small microbiota-derived metabolites from the essential amino acid tryptophan play an important role in host homeostasis. Some tryptophan metabolites have been reported to improve intestinal barrier function and protect against inflammation via the aryl hydrocarbon receptor^31^. Among the tryptophan metabolites, indole-3-lactic acid (ILA) was found in relatively higher levels in *Bifidobacterium* strains (e.g., *B. longum* subsp. *infantis*) isolated from the intestines of human infants compared with the other strains^18^. We have shown that breastmilk and specific probiotics, such as *Bifidobacteria*, can produce metabolic products that are anti-inflammatory and help protect the neonate from NEC^14–16^. Recently, we identified an efficient anti-inflammatory molecule in probiotics conditioned media (PCM) from *B. infantis* as ILA, a metabolite from breastmilk tryptophan^17^. ILA was reported as involved in inducing immunoregulatory T cells and suppressing inflammatory T cells, as well as possessing antioxidant and antimicrobial activity^32–35^. This was considered to be one of the benefits of normal maturation, including immune development in infants. Herein, transcription factors produced by ILA in immature enterocytes during inflammatory stress were identified, proving our hypothesis that ILA stimulates important genes that protect the neonatal intestine from inflammation and play a role in growth regulation. In addition, we noted that ILA also induces anti-viral gene expression in response to inflammation. Moreover, it is the first study to compare the different effects of ILA enterocyte mechanisms on anti-inflammation between immature and mature enterocytes both in vitro and in vivo*.*

Quantitative transcriptomic analysis, validated by real-time qRT-PCR, examined the gene transcriptional profile changes affected by ILA on an immature human small intestinal epithelial cell line (H4 cells) under normal and inflammatory conditions. ILA pretreatment had significant influence on negative regulation of the immune system process (Fig. 1c,d), suggesting the preventive effects of ILA on IL-1β-induced inflammatory stress in immature human enterocytes occurs via gene profile change (Figs. 1, 2). Our study also uncovered changes in a cluster pattern of genes which are associated with the interferon alpha/beta signaling pathway. In addition, signal transducers and activators of transcription1 (STAT1) were suggested to be the most important one among the key genes (Fig. 1e,f), suggesting a strong interaction with the partner gene CXCL10, also known as interferon gamma (IFN-γ) induced protein 10 (Table 2). STATs are a family of nuclear proteins mediating the action of a number of cytokines, hormones, and growth factors^36^. Among them, STAT1 is critical in the signal transduction pathway of IFN-γ and growth hormone, playing a major role in normal immune responses, particularly to viral, mycobacterial and fungal pathogens^37^. The primary function of IFN in the immune system is multifactorial, including protection from viral proliferation and macrophage activation to stimulate immune responses^38^. Yet IFN and its signaling molecules can affect interference with untoward inflammation^39^. For example, IFN and its STAT intermediates can regulate cell survival during various immune responses such as cytokine production, stimulation of antioxidants and apoptosis^40^. The above referenced studies underscore the role of IFN and STAT signaling molecules in mediated complex immune responses which may be detrimental to the host. In this study, we found that the STAT1 cell signaling pathway plays an important role in IL-1β-induced inflammation in H4 cells and ILA has a regulatory effect on the STAT1 signaling pathway in prevention of inflammation.

STAT1 has a critical role in innate immune responses associated with gene expression of many functional genes including anti-inflammatory, anti-viral and growth regulatory processes^28^. Our quantitative transcriptomic analysis demonstrated that STAT1 is a key gene in regulating the immune responses induced by ILA. Therefore, we speculated that the STAT1 cell signaling pathway plays an important role in IL-1β-induced inflammation in H4 cells and ILA has a regulatory effect on the STAT1 signaling pathway in the prevention of inflammation. Fludarabine phosphate (FAP) is an adenine nucleoside analogue that shows significant activity against chronic lymphocytic leukemia, indolent lymphoma, and acute leukemia, acting as a STAT-1 specific inhibitor^41,42^. In this study, FAP produced a consistent gene expression change in EPO, Tubulin-β3, Krt20, and RSAD2 compared with STAT1 (Figs. 6, S6), confirming the key role of the STAT1 cell signaling pathway in regulating an anti-inflammatory response. Interestingly, the STAT-1 specific inhibitor FAP did not decrease STAT-1 gene expression, but increased STAT-1 gene expression to inhibit IL-1β-induced excessive inflammation by the chemokine IL-8 gene expression and protein secretion in H4 cells. Studies linking regulation of chemokines by IFN/STAT with the establishment of immunity suggest this can benefit or weaken host immunity acting both as a driver or suppressor of intestinal inflammation in different diseases^40,43^. Herein, STAT1 obviously plays an anti-inflammatory role in immature enterocytes. In a manner similar to FAP, ILA could also inhibit IL-1β-induced intestinal inflammation by upregulating the STAT1 signaling pathway in immature enterocytes. ILA regulates the anti-inflammatory response via the STAT1 signaling pathway.

In addition, ILA had pleiotropic protective effects for immature enterocytes in inflammation, including anti-inflammatory, anti-viral, and cell growth potential both in vitro and in vivo (Figs. 2, 3, 4, 5, 6). ILA not only directly reduced the excessive inflammatory response (IL-8) to a stimulus (IL-1β), but also selectively induced a maturation of specific innate inflammatory response genes, including EPO, HBB, and STAT1. Erythropoietin was reported to have a range of actions beyond stimulation of erythropoiesis, promoting cell survival via activation of EPO receptors resulting in anti-apoptotic effects on ischemic tissues acting as a protective cytokine, protecting intestinal epithelial barrier function and thereby lowering the incidence of NEC in the immature intestine^23^. Functional EPO receptors are present in fetal and neonatal human and rat intestine, suggesting a physiological role of EPO in the developing gut^44^. Early erythroblasts are shown to possess immunosuppressive functions as a mechanism of inducing tolerance and allowing for commensal bacteria colonization in neonates. A cluster of immune-related genes becomes progressively upregulated as murine erythroblasts mature and are involved in the response to pathogenic infection. These include the STAT family, interferon, and other genes that are part of the innate immune response^45^. EPO was demonstrated to protect enterocyte barrier function by supporting expression of the tight junction (TJ) protein zonula occludens-1 (ZO-1) in the human fetal immature H4 intestinal epithelial cell line in vitro and preventing loss of ZO-1 in vivo, consequentially reversing the adverse effects of proinflammatory cytokines^23^. EPO also downregulated the inflammatory response of murine mast cells by dampening the release of proinflammatory cytokines IL-6 and TNF-α and therefore acting as an anti-inflammatory signal^46^. Moreover, EPO could upregulate HBB^24^ and increased fetal hemoglobin expression could improve osteoblast bone differentiation in association with decreased inflammatory cytokine release^47^. In addition, STAT1 was shown to be required for maintenance of stress erythropoiesis^48^. Our data showed a positive correlation with genes associated with an immune response. EPO, HBB, and STAT1 gene expression all were increased by ILA in human’s immature enterocyte H4 cells and immature mouse intestine C57-pup ileum during IL-1β inflammatory stimulation (Figs. 2, 3, 6).

Cytokines play a crucial rule in the development of inflammatory bowel injury during NEC^49^. In the gene profile data, we found that ILA pretreatment inhibited or reduced IL-1β induced plate-activating factor-1 (PAF1) (Fig. S7a), a PAF receptor (PAFR) mRNA (Fig. S7b), and TNF receptor associated factor2 (TRAF2) mRNA (Fig. S7d) expression. Both PAF and TRAF2 play an important role in mediating inflammatory injury in NEC development suggesting that ILA has an inhibitory effect on the mRNA expression of many cytokines. IL-10 is a protective cytokine, although ILA pretreatment increased IL-10 mRNA expression comparison with IL-1β stimulation. However, there is not a statistical significance.

In addition to the anti-inflammatory effects, ILA has anti-viral potential in response to inflammation and has regulatory effects on immature enterocyte growth. Two anti-viral genes IFIT2 and RSAD2 expression increased in ILA pretreated H4 cells and pup ileum in response to IL-1β stimulation (Fig. 4). IFIT2 and RSAD2 proteins are interferon-inducible proteins and have a viral inhibitory effect by functioning in the IFN-γ signaling pathway and innate immune system^26,27^. RSAD2 has an effect on a wide range of viruses by modulating cellular metabolic pathways that are essential for viral replication and/or cell proliferation and survival^27^. IFIT2 regulates the function of the cell cycle, apoptosis, tumor colonization and viral replication conferring cellular resistance to viral infections and regulating proliferation, apoptosis, and migration of cancer cells^50^. Among the transcription factors that bind to IFN-stimulated response elements and regulate IFN-induced expression of IFITs, the STAT1, STAT2, and IFN regulatory factor-9 plays a crucial role^51^. As an important downstream signaling molecule of IFNs, STAT1 has also been found to be dependent on innate immunity to defend against viral infection^52^. In addition, the C-terminal region of the hemoglobin β subunit HBB (112–147) was revealed to protect the fetus from viral and bacterial infections by forming a broadly active innate immune defense^25^. IFIT2 and STAT1 were both among the top 10 upregulated genes induced by ILA, highlighting a significant anti-viral potential for ILA. However, physiologic studies in vivo are needed to confirm this observation. Moreover, IFIT2 has been demonstrated to be a cytoskeleton-associated protein. Colocalization of IFIT2 and β-tubulin and enrichment of IFIT2 in the mitotic spindle were observed in cells undergoing mitosis which regulate cell growth^53^. The increase of IFIT2 and tubulin-β3 gene expression in H4 cells and pup-ileum pretreated with ILA (Figs. 4, 5), further suggests ILA’s benefit in regulatory immature enterocyte development.

ILA exhibited a differential anti-inflammatory, anti-viral, and developmental regulatory potential in immature versus mature human enterocytes under inflammatory stress (Figs. 2, 4, 5). Premature enterocytes respond to an inflammatory stimulus with excessive inflammation and can react to commensal bacteria with higher degrees of inflammation compared to mature human enterocytes^9,54^. In this study, IL-1β stimulation induced more than a 1500-fold IL-8 expression change in H4 cells compared to the control while less than a 40-fold change occurred in Caco-2 cells suggesting an excessive inflammatory response in immature enterocytes^9^. In H4 cells, ILA downregulates the excessive inflammatory chemokine IL-8 response and upregulates immune and growth regulation related genes EPO, HBB, IFIT2, RSAD2, STAT1, etc. However, these pleiotropic protective effects of ILA against inflammation were not noted in Caco-2 cells. A developmental difference was also noted between H4 cells and T84 cells in primary cultured mature human small intestinal epithelial cells^55^. Our previous reports have demonstrated a series of differences between probiotics on immune regulation of the inflammatory response, the endocytic process and barrier integrity among immature and mature human enterocytes^14–16,54,55^. It should be noted that ILA-induced maturation of specific innate immune response genes occurred only in immature and not in mature enterocytes, which is consistent with ILA as the source of *B. infantis* metabolites in premature infants and suggests a rationale for its effect on developmental regulation of the inflammatory response, confirming ILA was the efficient component of PCM that could developmentally regulate the intestinal innate immune response^14–16^.

In vivo, ILA affected anti-inflammatory, anti-viral, and developmental regulatory gene expression in a region-dependent and age-dependent manner in mouse intestine (Fig. 6). In a situation similar to immature and mature human enterocytes, developmental changes existed in immature only in immature mouse intestine. Okuyama et al.^56^ reported significant changes in the content and composition of intestinal mucus phospholipids during the first month of life. The increased incidence of bacterial translocation in newborns appears to be partially caused by the immaturity of intestinal mucosal barrier function. Interestingly, ILA only upregulated these innate immune response genes in immature mouse intestine (C57-pup-ileum). In contrast, ILA had little or no effects, and even completely opposite effects, on pup-colon or adult intestine. Ileal and colonic regional differences may be explained in terms of a different functional role of these two regions of the intestine and/or by a difference in origin of the sympathetic nerves supplying the two regions of the intestine^57^. A regional difference in streptozotocin-induced diabetes innervation, P-glycoprotein function, pathways for passive ion movement, stem and transit cell proliferation and apoptosis of the intestine have been reported previously^57–59^. In addition, the effects of ILA on embryonic and adult genes was different. HBB-y is hemoglobin beta-like embryonic chain, and HBB-b1 is hemoglobin beta adult major chain in mice, and hemoglobin switching from fetal to adult forms requires unique molecular regulators^60^. Keratins are a family of structural proteins that constitute the intermediate filaments of the cytoskeleton in intestinal epithelial cells. Krt18 is predominantly in the undifferentiated crypt compartment and Krt20 is predominantly detectable in the villus as a gastrointestinal differentiation marker^61,62^. HBB-y and Krt18, two embryonic genes that predominately exist in pup mice, are induced by ILA. In contrast, no remarkable change was found in adult major chains HBB-b1 and Krt20. The stimulation both in vitro and in vivo suggested that ILA benefited the immature intestine preferentially.

## Conclusion

Our observations in this study supported a new mechanistic model in which STAT1 acted as a key gene for ILA regulating innate immunity and cell development in immature enterocytes. As discussed above, our data illuminates a promising role for ILA interaction in the prevention and possible therapeutic potential for NEC in the premature infant. In addition, this STAT1-dependent anti-inflammatory activity establishes a link with anti-viral and developmental regulatory signaling which efficiently facilitates the pleiotropic benefit of ILA on intestinal immunity for immature enterocytes. Different protective reactions of ILA between immature and mature enterocytes also provides evidence that ILA anti inflammation is developmentally regulated. The ILA involvement of STAT1 gene transcription plays a significant role in maintaining immature intestinal health and increasing our current knowledge about what downstream intestinal epithelial signaling pathways encompass.

## Supplementary Information

Supplementary Information.

## Data Availability

All data are presented either in the main text or in the data supplement. The datasets generated and/or analyzed during the current study are available from the corresponding author on reasonable request.
