# Supplementary material for: The impact of indole-3-lactic acid on immature intestinal innate immunity and development: a transcriptomic analysis
Source: Sci Rep. 2021 Apr 13;11:8088. doi: 10.1038/s41598-021-87353-1 (PMC8044159; doi:10.1038/s41598-021-87353-1)
Supplement: Supplementary file 1 — Supplementary Information. [file 41598_2021_87353_MOESM1_ESM.pdf]

The impact of indole-3-lactic acid on immature intestinal innate immunity and development: A transcriptomic analysis

Wuyang Huang[1,3] , Ky Young Cho[2,3], Di Meng[3*] & W. Allan Walker[3*]

[1] Institute of Agro-Product Processing, Jiangsu Academy of Agricultural Sciences, Nanjing, PR China. [2]Department of Pediatrics, Kangnam Sacred Heart Hospital, Hallym University College of Medicine, Seoul, Korea. [3]Mucosal Immunology and Biology Research Center, Massachusetts General Hospital for Children, Harvard Medical School, Boston, Massachusetts, USA

Supplementary Information

Table S1. Primers for gene analysis.

Gene Name	Species		Primer sequences (5’-3’)
IL-8 (interleukin 8)	Human	forward	ACTGAGAGTGATTGAGAGTGGAC
		reverse	AACCCTCTGCACCCAGTTTTC
STAT1 (signal transducer and activator of transcription 1)	Human	forward	CGGCTGAATTTCGGCACCT
		reverse	CAGTAACGATGAGAGGACCCT
EPO (erythropoietin)	Human	forward	GGAGGCCGAGAATATCACGAC
		reverse	CCCTGCCAGACTTCTACGG
CDA (cytidine deaminase)	Human	forward	CTGAACGGACCGCTATCCAG
		reverse	AGGCCCCACATGGAGAGATAA
HBB (hemoglobin beta)	Human	forward	CGGGTCCGCCTATACTTCTTC
		reverse	CGTAGGGCAGGAGTTTCAGG
IFIT2 (interferon-induced protein with tetratricopeptide repeats 2)	Human	forward	GACACGGTTAAAGTGTGGAGG
		reverse	TCCAGACGGTAGCTTGCTATT
RSAD2 (radical S-adenosyl methionine domain containing 2)	Human	forward	TTGGACATTCTCGCTATCTCCT
		reverse	AGTGCTTTGATCTGTTCCGTC
INFγ (interferon gamma)	Human	forward	TCGGTAACTGACTTGAATGT
		reverse	TCGCTTCCCTGTTTTAGCTGC
CYP1A1 (cytochrome P450 family 1 subfamily A polypeptide 1)	Human	forward	ACATGCTGACCCTGGGAAAG
		reverse	GGTGTGGAGCCAATTCGGAT
Tubulin-β3	Human	forward	GGCCAAGGGTCACTACACG
		reverse	GCAGTCGCAGTTTTCACACTC
Krt18 (keratin 18)	Human	forward	GTTGACCGTGGAGGTAGATGC
		reverse	GAGCCAGCTCGTCATATTGGG

Krt20 (keratin 20)	Human	forward	GGACGACACCCAGCGTTTAT
		reverse	CGCTCCCATAGTTCACCGTG
Ki67	Human	forward	GGGCCAATCCTGTCGCTTAAT
		reverse	GTTATGCGCTTGCGAACCT
GAPDH (glyceraldehyde 3-phosphate dehydrogenase)	Human	forward	ATGGGGAAGGTGAAGGTCG
		reverse	GGGGTCATTGATGGCAACAATA
STAT1 (signal transducer and activator of transcription 1)	Mouse	forward	GCTGCCTATGATGTCTCGTTT
		reverse	TGCTTTTCCGTATGTTGTGCT
EPO (erythropoietin)	Mouse	forward	CCTCATCTGCGACAGTCGAG
		reverse	ACAACCCATCGTGACATTTTCT
CDA (cytidine deaminase)	Mouse	forward	AGGGCTATTGCCATCTCTAGTG
		reverse	CCAGTCGGTGCCAAACTCTC
HBB-y (hemoglobin Y, beta-like embryonic chain)	Mouse	forward	TGGCCTGTGGAGTAAGGTCAA
		reverse	GGGTTGCCCATTATGGCAGA
HBB-b1(hemoglobin beta adult major chain)	Mouse	forward	GCACCTGACTGATGCTGAGAA
		reverse	TTCATCGGCGTTCACCTTTCC
IFIT2 (interferon-induced protein with tetratricopeptide repeats 2)	Mouse	forward	AGAACCAAAACGAGAGAGTGAAG
		reverse	TCCAGACGGTAGTTCGCAATG
RSAD2 (radical S-adenosyl methionine domain containing 2)	Mouse	forward	AGCATTAGGGTGGCTAGATCC
		reverse	CTGAGTGCTGTTCCCATCTTC
Tubulin-β3	Mouse	forward	GCGCCTTTGGACACCTATTCA
		reverse	GCCCTCCGTATAGTGCCCT
Krt18 (keratin 18)	Mouse	forward	CAGCCAGCGTCTATGCAGG
		reverse	CCTTCTCGGTCTGGATTCCAC
Krt20 (keratin 20)	Mouse	forward	CAACGGATCGGACCTGTTTG
		reverse	AGCGCACTTTTTCTAGGTAGTTT
Ki67	Mouse	forward	GGGCCAATCCTGTCGCTTAAT
		reverse	GTTATGCGCTTGCGAACCT

GAPDH (glyceraldehyde 3-phosphate dehydrogenase)	Mouse	forward	ATGACCTTGCCCACAGCCT
		reverse	CCTGCACCACCAACTGCTTA

Table S2. Overlapping Enriched KEEG Pathways for DEGs in the IL-1β/con, ILA-IL-1β /con, and ILA/con groups.

**KEGG Pathways for DEGs**	**IL-1β /con**	**ILA-IL-1β /con**	**ILA /con**
Intrinsic Pathway for Apoptosis	3.E-06	3.E-02	
Toll Like Receptor 4 (TLR4) Cascade	3.E-06	9.E-08	
Activated TLR4 signalling	1.E-06	4.E-08	
MyD88:Mal cascade initiated on plasma membrane	1.E-03	1.E-04	
MyD88-independent TLR3/TLR4 cascade	5.E-07	1.E-10	
Signalling by NGF	4.E-02	1.E-07	
Toll Like Receptor 9 (TLR9) Cascade	1.E-03	0.E+00	
Toll Like Receptor 10 (TLR10) Cascade	1.E-03	0.E+00	
Toll Like Receptor 3 (TLR3) Cascade	5.E-07	1.E-10	
Toll Like Receptor 5 (TLR5) Cascade	1.E-03	0.E+00	
Toll Like Receptor TLR1:TLR2 Cascade	1.E-03	1.E-04	
TRAF6 Mediated Induction of proinflammatory cytokines	1.E-03	8.E-07	
Toll Like Receptor 7/8 (TLR7/8) Cascade	8.E-04	0.E+00	
Toll Like Receptor TLR6:TLR2 Cascade	1.E-03	1.E-04	
NOD1/2 Signaling Pathway	4.E-03	8.E-03	
Nucleotide-binding domain, leucine rich repeat containing receptor (NLR) signaling pathways	2.E-03	5.E-02	
Toll-Like Receptors Cascades	1.E-05	1.E-06	

RIG-I/MDA5 mediated induction of IFN-alpha/beta pathways	2.E-03	0.E+00	
APC/C-mediated degradation of cell cycle proteins	2.E-02		6.E-04
APC/C:Cdh1 mediated degradation of Cdc20 and other APC/C:Cdh1 targeted proteins in late mitosis/early G1	3.E-02		3.E-02
Regulation of APC/C activators between G1/S and early anaphase	2.E-02		4.E-02
APC/C:Cdc20 mediated degradation of mitotic proteins	2.E-02		3.E-02
Activation of APC/C and APC/C:Cdc20 mediated degradation of mitotic proteins	2.E-02		8.E-03
Toll Like Receptor 2 (TLR2) Cascade	1.E-03	1.E-04	
NGF signalling via TRKA from the plasma membrane	2.E-02	0.E+00	
Signaling by FGFR	5.E-02	7.E-04	
TAK1 activates NFkB by phosphorylation and activation of IKKs complex	4.E-04	1.E-04	
Interleukin-1 signaling	2.E-03	7.E-03	
Signaling by Interleukins	2.E-04	2.E-03	
MAP kinase activation in TLR cascade	1.E-02	2.E-04	
activated TAK1 mediates p38 MAPK activation	2.E-02	2.E-02	
Regulation of mitotic cell cycle	2.E-02		6.E-04
Interferon gamma signaling	1.E-04	0.E+00	
Interferon alpha/beta signaling	1.E-04	2.E-06	
Interferon Signaling	0.E+00	3.E-06	
Negative regulators of RIG-I/MDA5 signaling	3.E-04	1.E-03	

TRIF-mediated TLR3/TLR4 signaling	4.E-07	1.E-10
TRAF6 mediated induction of NFkB and MAP kinases upon TLR7/8 or 9 activation	1.E-03	0.E+00
MyD88 dependent cascade initiated on endosome	8.E-04	0.E+00
MyD88 cascade initiated on plasma membrane	1.E-03	0.E+00
Signaling by the B Cell Receptor (BCR)	2.E-03	6.E-04
Downstream signaling events of B Cell Receptor (BCR)	2.E-04	8.E-04
Signaling by ERBB2	5.E-02	4.E-04
Cytokine Signaling in Immune system	1.E-08	4.E-09
Adaptive Immune System	4.E-02	4.E-02
Signaling by SCF-KIT	5.E-02	0.E+00
Cellular responses to stress	2.E-02	3.E-03
Fc epsilon receptor (FCERI) signaling	1.E-02	2.E-06
FCERI mediated NF-κB activation	2.E-02	
Diseases of Immune System	4.E-02	5.E-02
Diseases associated with the TLR signaling cascade	4.E-02	5.E-02
Signaling by FGFR1	5.E-02	7.E-04
Signaling by FGFR2	5.E-02	7.E-04
Signaling by FGFR3	5.E-02	7.E-04
Signaling by FGFR4	5.E-02	7.E-04

Values are *P* value adjusted by benjamini-hochberg FDR multiple testing.

Figure S1.

(a)

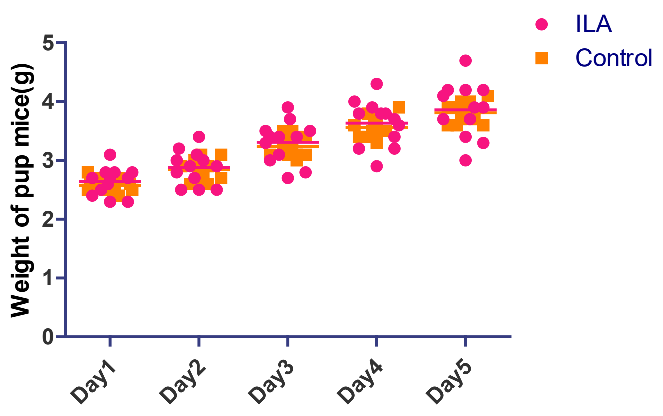

(b)

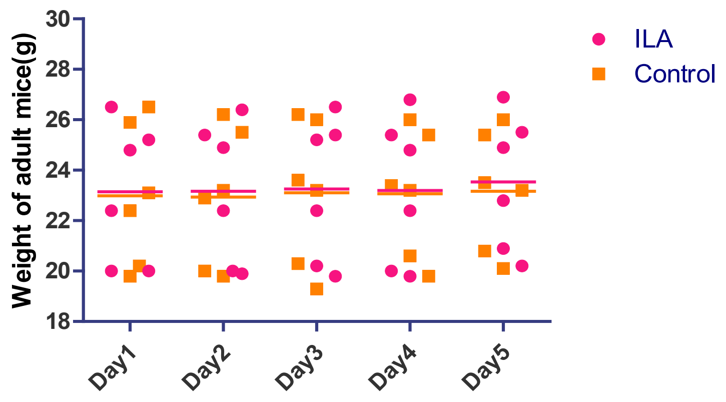

Figure S1. The weights of C57BL/6J pup mice (**a**, n=11-12) and adult mice (**b**, n=6) treated with or without 10 μM ILA.

Figure S2.

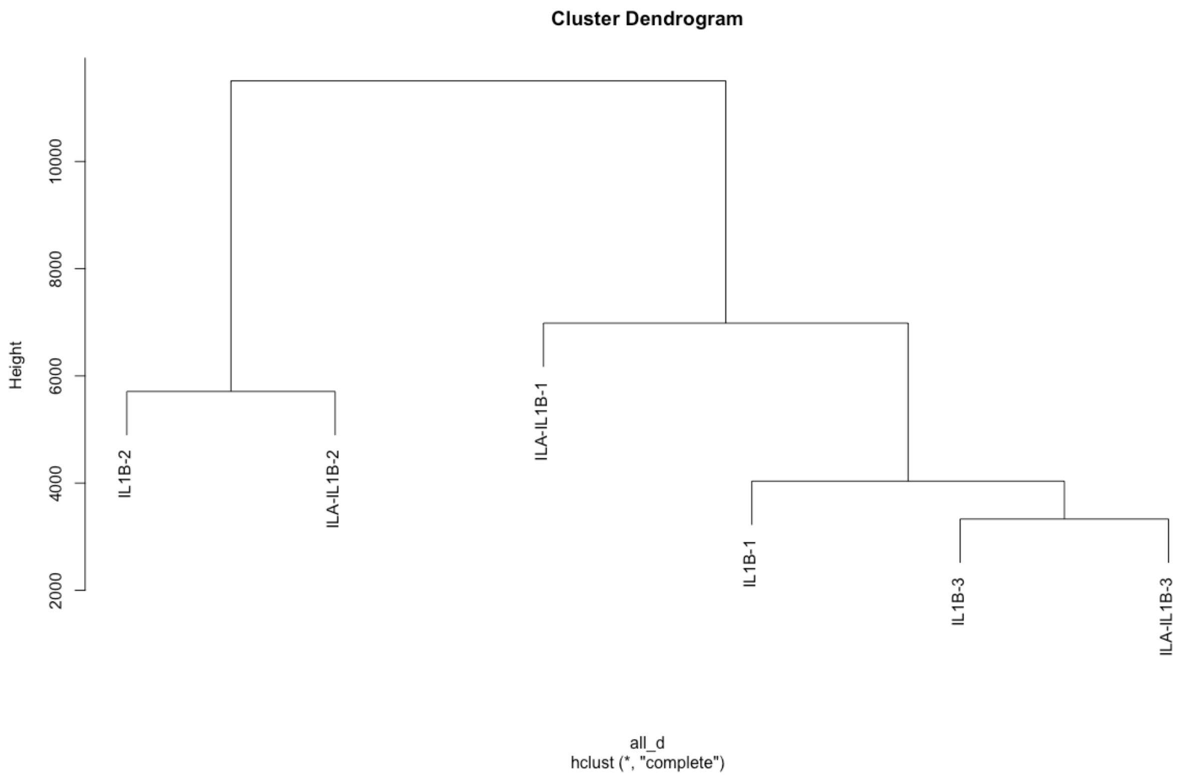

Figure S2. Hierarchical clustering analysis in the triplet samples of the control, IL-1β, and ILA-IL-1β groups.

Figure S3.

(a)

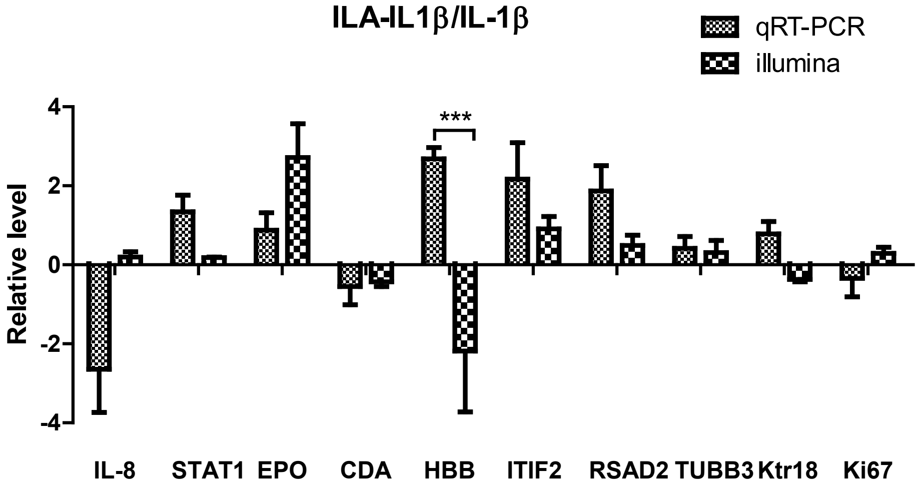

(b)

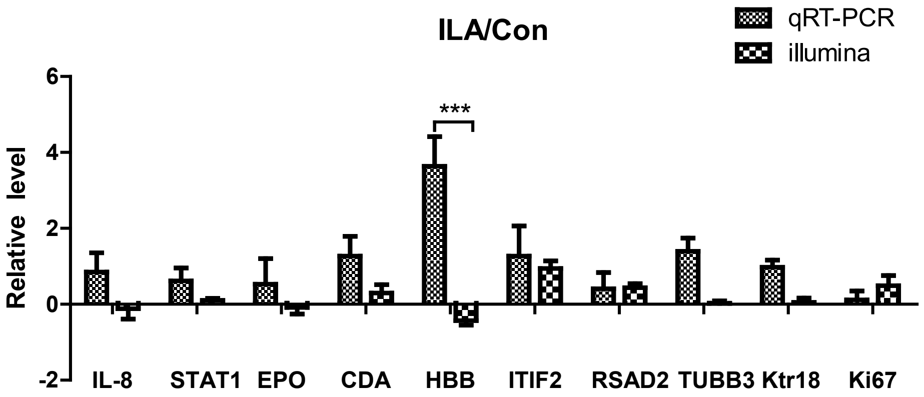

Figure S3. Comparison of (**a**) ILA-IL-1β/IL-1β and (**b**) ILA/control group gene expression change ratio that were obtained by qRT-PCR and RNA-sequencing Illumina assay.

Figure S4.

(a)

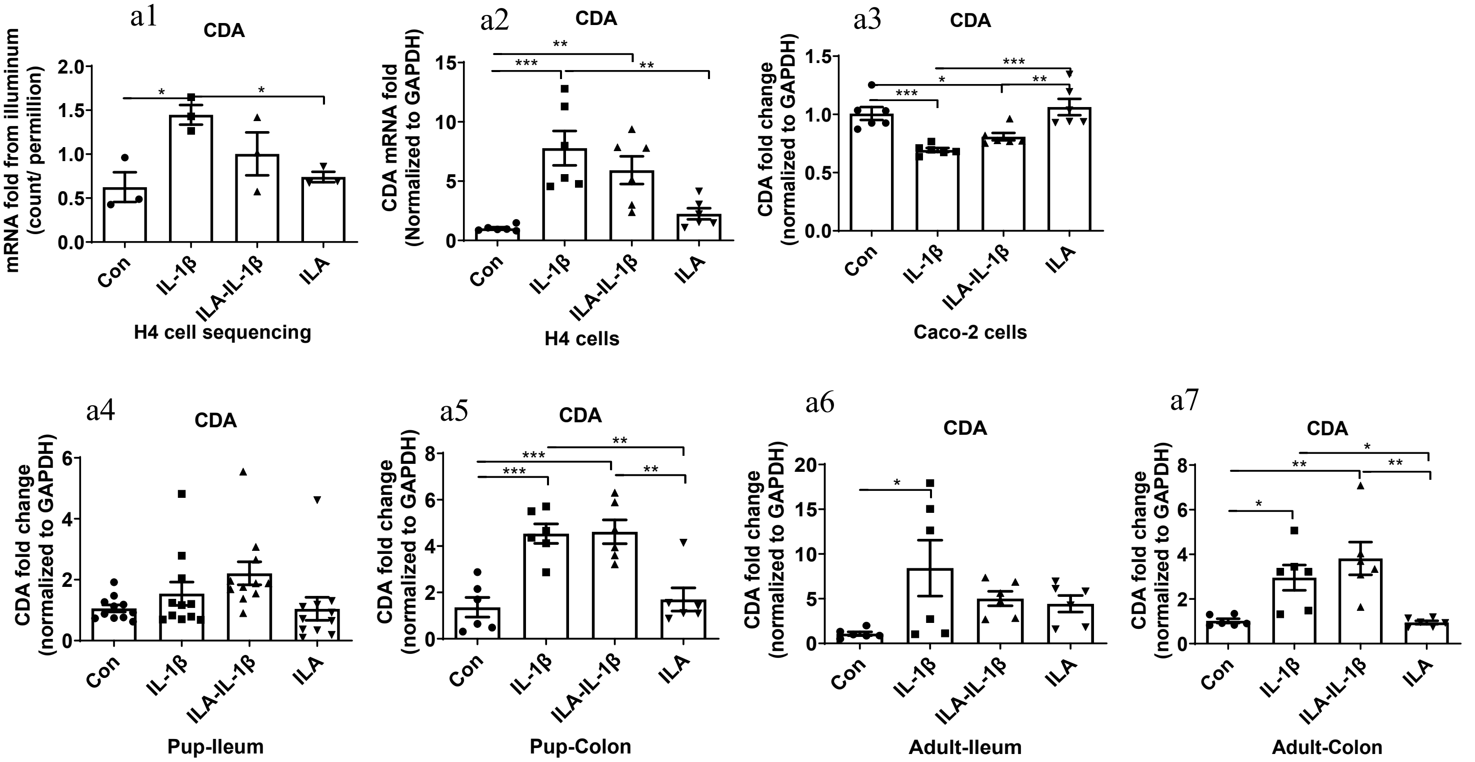
a1
CDA
mRNA fold from illuminum (count/ permillion)
2.0
1.5
1.0
0.5
0.0
*
*
Con
IL-1β
ILA-IL-1β
ILA
H4 cell sequencing
a2
CDA
CDA mRNA fold (Normalized to GAPDH)
15
10
5
0
**
***
**
Con
IL-1β
ILA-IL-1β
ILA
H4 cells
a3
CDA
CDA fold change (normalized to GAPDH)
1.5
1.0
0.5
0.0
***
*
**
***
Con
IL-1β
ILA-IL-1β
ILA
Caco-2 cells
a4
CDA
CDA fold change (normalized to GAPDH)
6
4
2
0
Con
IL-1β
ILA-IL-1β
ILA
Pup-Ileum
a5
CDA
CDA fold change (normalized to GAPDH)
8
6
4
2
0
***
**
***
**
Con
IL-1β
ILA-IL-1β
ILA
Pup-Colon
a6
CDA
CDA fold change (normalized to GAPDH)
20
15
10
5
0
*
Con
IL-1β
ILA-IL-1β
ILA
Adult-Ileum
a7
CDA
CDA fold change (normalized to GAPDH)
8
6
4
2
0
*
**
**
*
Con
IL-1β
ILA-IL-1β
ILA
Adult-Colon

(b)

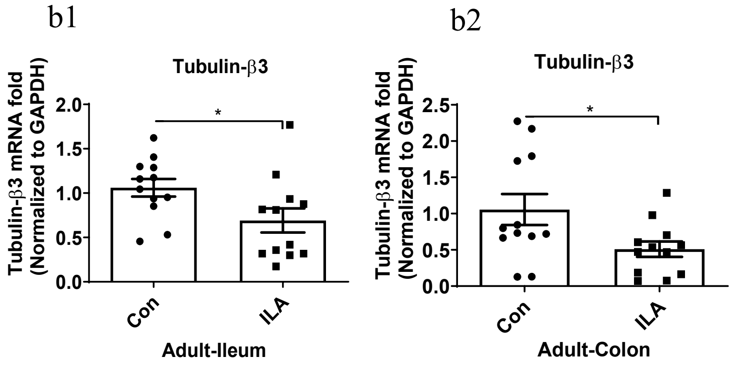
b1
Tubulin-β3
Tubulin-β3 mRNA fold (Normalized to GAPDH)
2.0
1.5
1.0
0.5
0.0
*
Con
ILA
Adult-Ileum
b2
Tubulin-β3
Tubulin-β3 mRNA fold (Normalized to GAPDH)
2.5
2.0
1.5
1.0
0.5
0.0
*
Con
ILA
Adult-Colon

(c)

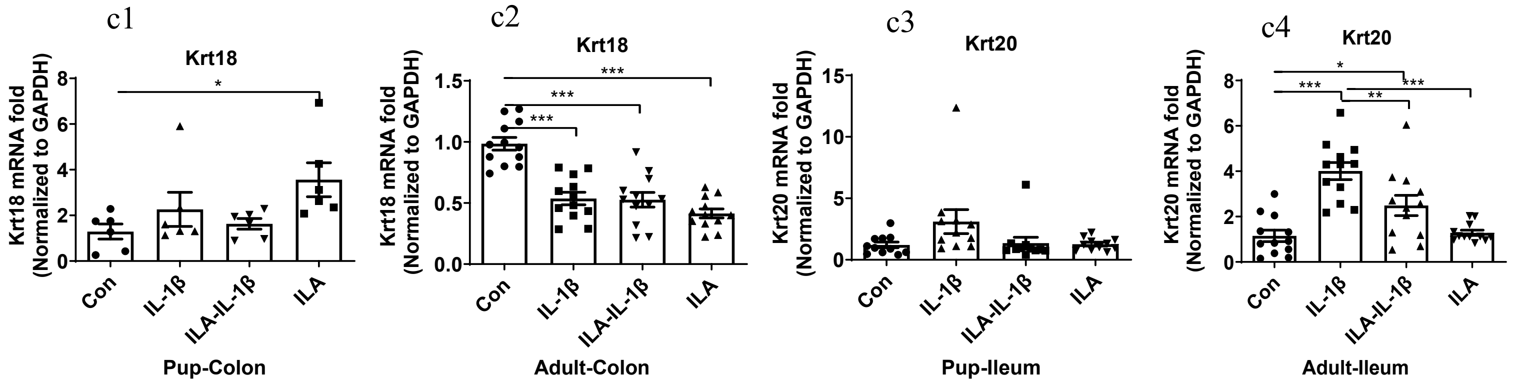

(d)

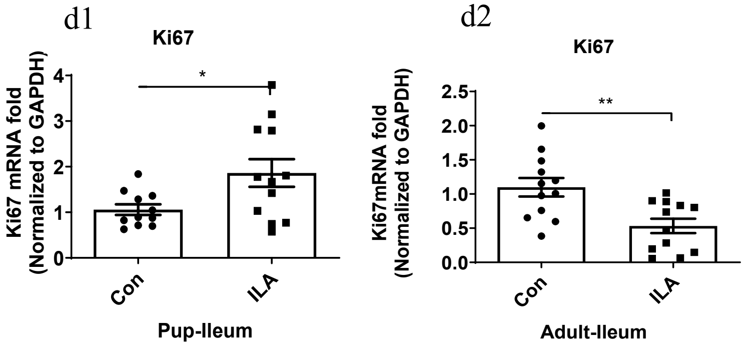

Figure S4. The effects of ILA on cell growth related genes. (**a**) cytidine deaminase CDA mRNA level analyzed by RNA sequencing using Illumina (a1) and validated by qRT PCR in H4 cells (a2). CDA mRNA expression in Caco-2 cells (a3), pup-ileum (a4) and colon (a5), adult ileum (a6) and colon (a7) were determined by qRT-PCR; (**b**) Tubulin-β3 mRNA expression in adult ileum (b1) and colon (b2); (**c**) embryonic keratin Krt 18 mRNA expression in pup colon (c1) and adult colon (c2), and adult keratin Krt20 mRNA expression in pup ileum (c3) and adult ileum (c4); and (**d**) Ki67 mRNA expression in pup ileum (d1) and adult ileum (d2). H4 or Caco-2 cells were pretreated with ILA (5 μM) for 24 hrs before IL-1β stimulation (1 ng/mL for 4 hrs). Pup or adult mice were fed with or without ILA (10 μM) for 5 days, whose intestinal tissues were incubated with or without IL-1β stimulation (1 ng/mL) for 2 hrs. Data are represented as the mean ± SEM (n=3-12). One-way ANOVA and Tukey post hoc tests were used for statistical analysis. Differences were considered significant at *$p < 0.05$, **$p < 0.01$, *** $p < 0.001$.

Figure S5.

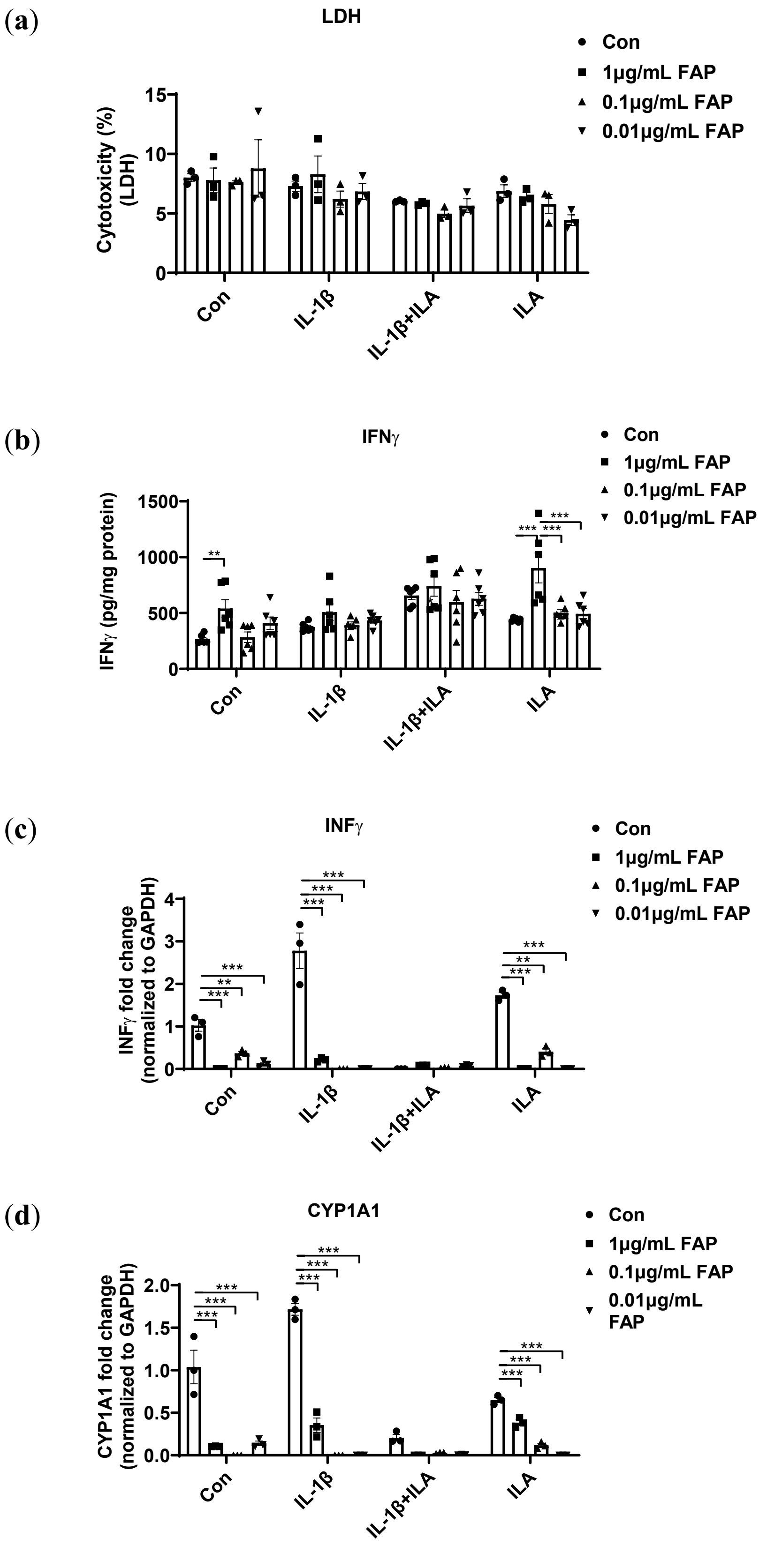
(a)
LDH
Cytotoxicity (%) (LDH)
Con
IL-1β
IL-1β+ILA
ILA
Con
1μg/mL FAP
0.1μg/mL FAP
0.01μg/mL FAP
(b)
IFNγ
IFNγ (pg/mg protein)
(c)
INFγ
INFγ fold change (normalized to GAPDH)
(d)
CYP1A1
CYP1A1 fold change (normalized to GAPDH)

Figure S5. The effects on different concentration of STAT1 inhibitor FAP on ILA pretreated IL-1β-stimulated H4 cells. (**a**) LDH cytotoxicity and (**b**) interferon gamma INFγ secretion in H4 supernatant; (**c**) INFγ and (**d**), cytochrome P450 family 1 subfamily A polypeptide 1 (CYP1A1) mRNA expression in H4 cells. Different concentration of STAT1 inhibitor FAP (1, 0.1, and 0,01 μg/mL) were pretreated 30 min before ILA (5 μM) treatment for 24 hrs then exposed to IL-1β (1 ng/mL for 24 hrs for ELISA or 4 hrs for mRNA analysis). The secretion of INFγ into the cell culture supernatant was determined by ELISA, while mRNA was analyzed by qRT PCR. Data are represented as the mean ± SEM (n=6). Two-way ANOVA were used for statistical analysis. Differences were considered significant at $^{**}p < 0.01$ and $^{***}\ p < 0.001$.

Figure S6.

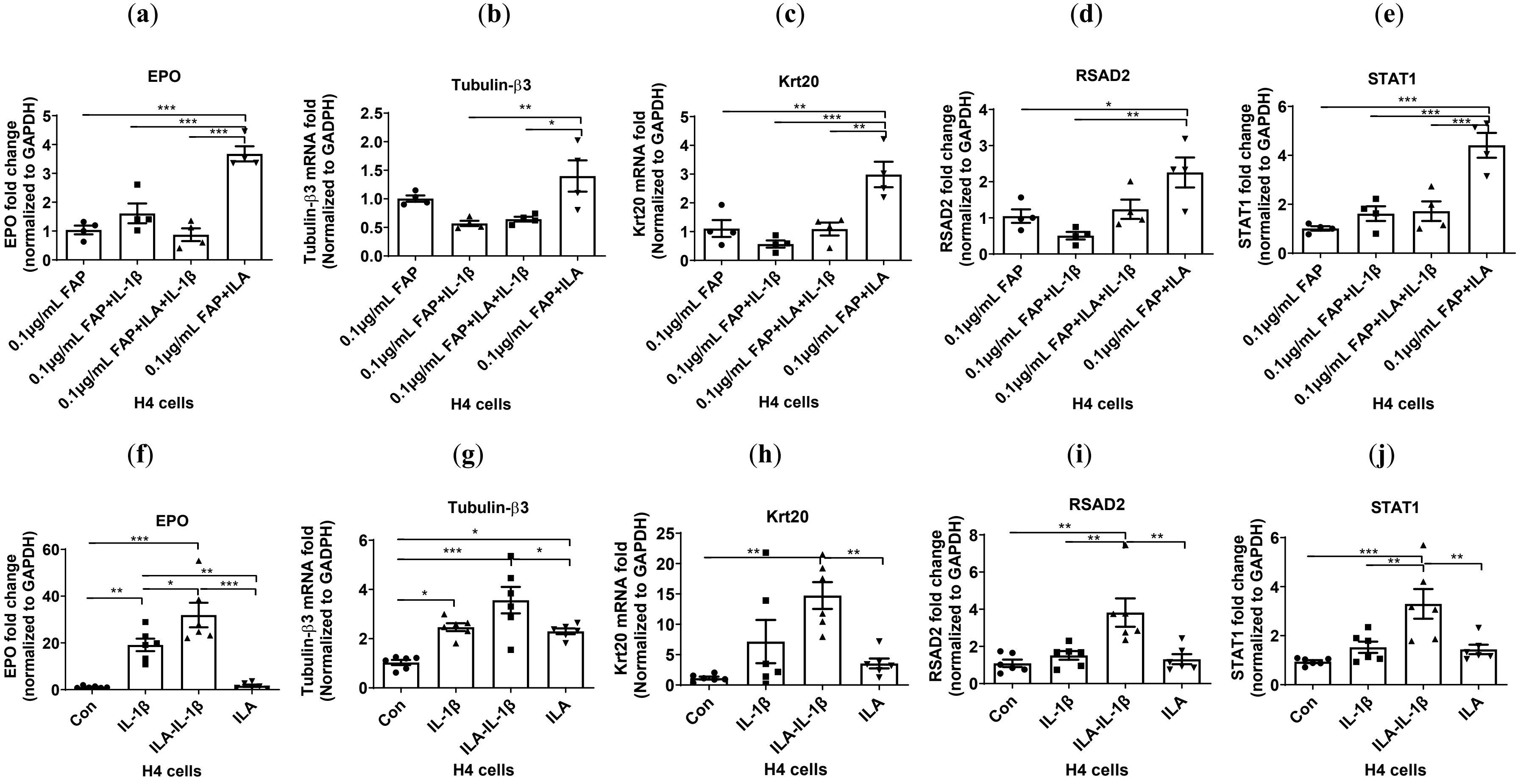

(a)
(b)
(c)
(d)
(e)
EPO
EPO fold change (normalized to GAPDH)
Tubulin-β3
Tubulin-β3 mRNA fold (Normalized to GAPDH)
Krt20
Krt20 mRNA fold (Normalized to GAPDH)
RSAD2
RSAD2 fold change (normalized to GAPDH)
STAT1
STAT1 fold change (normalized to GAPDH)
0.1μg/mL FAP
0.1μg/mL FAP+IL-1β
0.1μg/mL FAP+ILA+IL-1β
0.1μg/mL FAP+ILA
H4 cells
(f)
(g)
(h)
(i)
(j)
Con
IL-1β
ILA-IL-1β
ILA

Figure S6. Comparison the effects on different genes in ILA pretreated IL-1β-stimulated H4 cells with or without STAT1 inhibitor FAP. (**a**) EPO, (**b**) Tubulin-β3, (**c**) Krt20, (**d**) RSAD2, and (**e**) STAT1 mRNA expression level of qRT PCR in H4 cells (FAP *vs* Con). FAP (0.1 μg/mL) was pretreated 30 min before ILA (5 μM) treatment for 24 hrs then exposed to IL-1β (1 ng/mL for 4 hrs). Data are represented as the mean ± SEM (n=6). One-way ANOVA and Tukey post hoc tests were used for statistical analysis. Differences were considered significant at *$p < 0.05$, **$p < 0.01$, *** $p < 0.001$. Abbreviations: STAT1, signal transducer and activator of transcription 1; FAP, fludarabin phosphate; EPO, erythropoietin; Krt20, keratin 20; RSAD2, radical S-adenosyl methionine domain containing 2.

Figure S7.

(a)

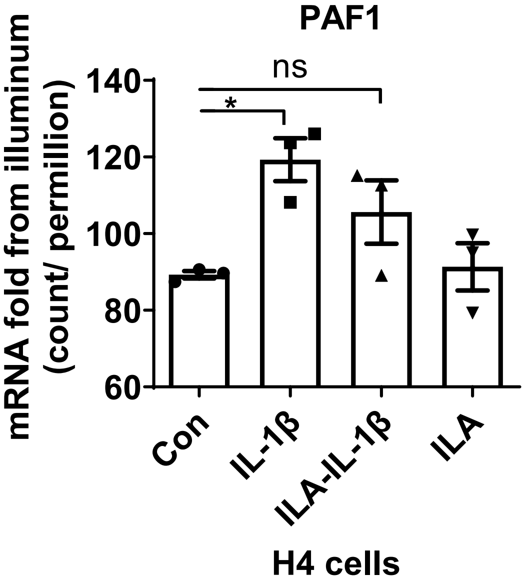
PAF1
mRNA fold from illuminum
(count/ permillion)
140
120
100
80
60
ns
*
Con
IL-1β
ILA-IL-1β
ILA
H4 cells

(b)

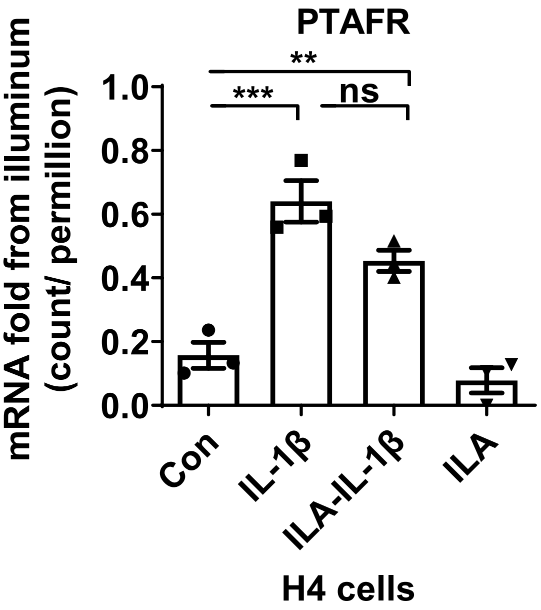
PTAFR
mRNA fold from illuminum
(count/ permillion)
1.0
0.8
0.6
0.4
0.2
0.0
**
***
ns
Con
IL-1β
ILA-IL-1β
ILA
H4 cells

(c)

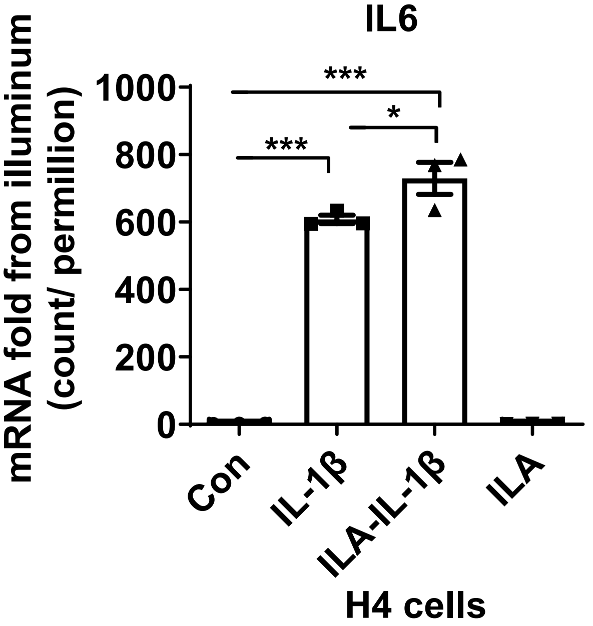
IL6
***
*
***
mRNA fold from illuminum (count/ permillion)
1000
800
600
400
200
0
Con
IL-1β
ILA-IL-1β
ILA
H4 cells

(d)

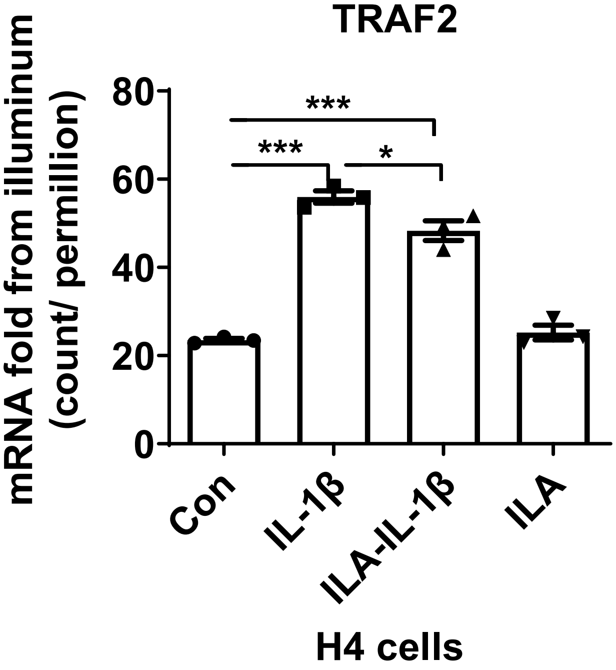
TRAF2
***
***
*
mRNA fold from illuminum (count/ permillion)
80
60
40
20
0
Con
IL-1β
ILA-IL-1β
ILA
H4 cells

(e)

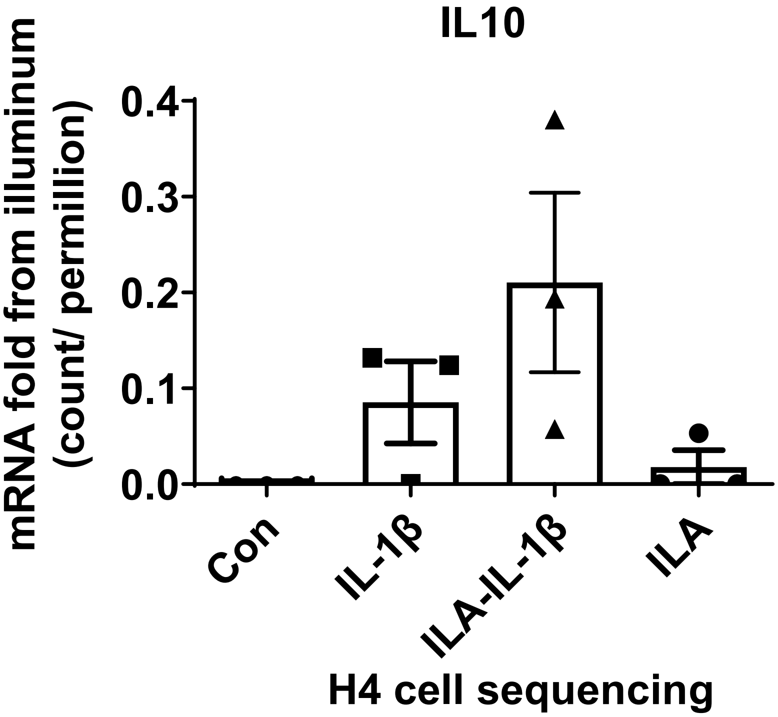

Figure S7. The gene change of PAF, IL6, TNFα and IL-10 cells in response to IL-1β stimulation in immature enterocytes H4 cells. H4 cells were pretreated with ILA before IL-1β stimulation, the mRNA levels were detected by RNA sequencing illumina assay. Data are represented as the mean ± SEM (n=3). One-way ANOVA and Tukey post hoc tests were used for statistical analysis. Differences were considered significant at *$p < 0.05$, **$p < 0.01$, *** $p < 0.001$. Abbreviations: PAF1, Plate-activating factor-1; PTAFR, platelet-activating factor receptor; IL6, interleukin 6; IL6R, interleukin 6 receptor; TRAF2, TNF receptor associated factor2; IL10, interleukin 10; IL-10Rβ, interleukin 10 receptor β.
